# Functional Analysis of the Cortical Transcriptome and Proteome Reveal Neurogenesis, Inflammation, and Cell Death after Repeated Traumatic Brain Injury *In vivo*

**DOI:** 10.1089/neur.2021.0059

**Published:** 2022-06-13

**Authors:** Celeste S. Dunn, Laís A. Ferreira, Sara M. Venier, Syed F. Ali, Jeffrey C. Wolchok, Kartik Balachandran

**Affiliations:** ^1^ Cell and Molecular Biology Program, University of Arkansas, Fayetteville, Arkansas, USA; ^2^ Department of Biological Sciences, University of Arkansas, Fayetteville, Arkansas, USA; ^3^ Neurochemistry Laboratory, Division of Neurotoxicology, National Center for Toxicological Research, Jefferson, Arkansas, USA.; ^4^ Center for Integrativve Nanotechnology Sciences, University of Arkansas at Little Rock, Little Rock, Arkansas, USA.; ^5^ Department of Biomedical Engineering, University of Arkansas, Fayetteville, Arkansas, USA

**Keywords:** proteome, repeated TBI, TBI, transcriptome

## Abstract

The pathological effects of repeated traumatic brain injuries (TBIs) are largely unknown. To gain a detailed understanding of the cortical tissue acute biological response after one or two TBIs, we utilized RNA-sequencing and protein mass spectrometry techniques. Using our previously validated C57Bl/6 weight-drop model, we administered one or two TBIs of a mild or moderate severity. Double injury conditions were spaced 7 days apart, and cortical tissue was isolated 24 h after final injury. Analysis was carried out through functional gene annotation, utilizing Gene Ontology, for both the proteome and transcriptome. Major themes across the four different conditions include: neurogenesis; inflammation and immune response; cell death; angiogenesis; protein modification; and cell communication. Proteins associated with neurogenesis were found to be upregulated after single injuries. Transcripts associated with angiogenesis were upregulated in the moderate single, mild double, and moderate double TBI conditions. Genes associated with inflammation and immune response were upregulated in every condition, with the moderate single condition reporting the most functional groups. Proteins or genes involved in cell death, or apoptosis, were upregulated in every condition. Our results emphasize the significant differences found in proteomic and transcriptomic changes in single versus double injuries. Further, cortical omics analysis offers important insights for future studies aiming to deepen current knowledge on the development of secondary injuries and neurobehavioral impairments after brain trauma.

## Introduction

Traumatic brain injury (TBI) can lead to deficits in cognitive, physical, and/or psychosocial functions—potentially causing permanent damage.^1^ In the United States, TBIs are responsible for >2.8 million emergency department visits and hospitalizations—of which >58,000 are fatal.^2^ There are limited treatment options for TBIs because the pathophysiology of secondary injuries are varied and not well characterized. Studies have shown that the symptoms and cognitive impairment resulting from TBI may last anywhere from 1 week to up to 3 months.^3^ This is relevant when we consider the impact of repetitive TBIs in a time frame in which the brain has not fully recovered from previous injuries.

Whereas a single injury can have severe outcomes, repeated TBIs can compound these effects.^4–6^ Studies have shown that repeated injuries in humans can lead to memory impairment and cognitive deficits.^7,8^ Those who suffer from repeated TBIs are also more likely to experience depression later in life than those who suffer one injury,^9^ and animal models have shown that repeated TBIs experienced earlier in life can lead to delayed development and lasting behavioral deficits.^10^ Additionally, repeated injuries also increase the likelihood of neurodegenerative diseases such as Alzheimer's and Parkinson's diseases.^11,12^

Although the clinical effects of repeated TBIs are more established, there is a limited understanding of the acute molecular responses and associated biological processes potentiated by repeated TBIs. It is known that repeated injuries lead to neurodegeneration, long-term neuroinflammation, and apoptosis.^13,14^ Further, angiogenesis, cerebral edema, and long-term white matter disruption are also present after repeated injuries.^15,16^ Identifying the presence of these secondary effects after repeated TBI provides broad observations; however, a more comprehensive understanding of the entire cellular response is needed to identify potential therapeutic targets for the development of efficient treatments for patients with repeated TBI.

To address the above gaps, we analyzed the cortical transcriptome and proteome of a C57Bl/6 mouse model after repeated injury. Transcriptomics- and proteomics-based approaches can provide an exhaustive understanding of the molecular response of the brain to injury, leading to insights that can contribute to a better understanding of the mechanisms involved in secondary injuries.^17–20^ One or two, mild or moderate, TBIs, spaced 7 days apart, were administered and the cortical tissue was analyzed 24 h after final injury. Functional annotation was performed on the omics data using Gene Ontology (GO).^21,22^ From our analysis, we conclude that: 1) neurogenesis was upregulated after single injuries, 2) inflammation was upregulated after all injuries, and 3) cell death was upregulated in the moderate and double injury conditions.

## Methods

### Animal procedures

All procedures involving mice in this study were approved by the University of Arkansas Institutional Animal Care and Use Committee. Male 6-week-old C57BL/6 mice (The Jackson Laboratory, Bar Harbor, ME) were randomly sorted into control and injured groups. Animals were subjected to daily general health, mortality, and morbidity assessments, and no differences between TBI- and sham-treated animals were observed. TBI was induced using our published closed-head model, and post-injury care was carried out accordingly.^23^ Control mice were given a single sham TBI or double sham TBI. Injured mice were given a mild single (MiS), mild double (MiD), moderate single (MoS), or moderate double TBI (MoD). A g-force (78.6 ± 10.3) was used to deliver a mild TBI and 137.4 ± 9.6 g-force for a moderate TBI.^23^ Although the sham mice were not subjected to TBI, they underwent the same anesthesia protocol and medication regimen, once for the single impact control and twice, with a 7-day interval, for the double injury control. All mice were euthanized 24 h after final or sham TBI. After euthanasia, brains were immediately dissected and washed in phosphate-buffered saline. Olfactory bulb, cerebellum, and pons were discarded, and the pooled cortex, thalamus, hippocampus, and midbrain were flash frozen in liquid nitrogen for RNA and protein extraction.

### RNA sequencing and analysis

To isolate RNA, TRIzol (Invitrogen, Grand Island, NY) was added to the frozen samples, tissue was homogenized, and chloroform was added for phase separation (RNeasy Mini Kit; Qiagen, Germantown, MD). RNA samples (RNA integrity number, >7.0; 28S/18S, >2.0) were analyzed by RNA-sequencing (RNA-seq) on the BGISeq-500 platform. Mean depth read was 20,000,000 reads per complementary DNA library. RNA-seq reads were processed with FastqGroomer (version 1.1.5) and mapped to the reference genome, *Mus musculus* (mm10), with RNA Star (version 2.6.0).^13,14^ Binary alignment map files were further analyzed with FeatureCounts. edgeR was then used to perform differential gene expression analysis, using a cutoff value of 1 CPM to filter low-count transcripts.^15,16^ Significance of differential gene expression values was performed using edgeR with normalization to respective single or double control. A sample size of 7 was used for each of the six conditions.

### Protein collection and sequencing

To isolate the protein samples, flash-frozen tissue was homogenized in radioimmunoprecipitation assay lysis buffer (Santa Cruz Biotechnology, Santa Cruz, CA) and centrifuged at 13,000*g* for 5 min at 4°C. The supernatant was collected and purified before digestion with trypsin. Peptides were separated on a column and eluted. Eluted peptides were ionized by electrospray, followed by mass spectrometric analysis, at the IDeA Proteomics Facility. The chromatogram library was assembled, and quantitative analysis was performed to obtain a comprehensive proteomic profile. Proteins were quantified and identified using EncyclopeDIA, with 1% false discovery thresholds used at both the protein and peptide levels.^24^ Protein quality was assessed using an in-house ProteiNorm app.^25^ Data were normalized using cyclic loess. A sample size of 4 was used for each of the six conditions.

### Data and statistical analysis

All RNA-seq data were deposited in the NCBI SRA database (PRJNA664018). A file containing all the transcriptomics and proteomics log_2_ fold-change data, as well as the respective *p* and *q* values, was deposited on GitHub.^25^ Heatmaps were created using Morpheus (https://software.broadinstitute.org/morpheus), and the mixOMICS R package was used to determine the effects of TBI on protein and transcript expression levels.^27^ Transcript data were filtered to include at least 100 gene counts in each sample, and the proteome data were not filtered. Graphs were created on Prism software (version 8; GraphPad Software Inc., La Jolla, CA). For individual analysis, samples from 7 animals were used for transcriptomics and 4 for proteomics, whereas comparisons between both were performed using matched tissues from the same 4 animals. Statistical significance was defined as *p* < 0.05.

### Functional annotation and clustering

The lists of transcripts and proteins that had their expression levels significantly altered after TBI (*p* < 0.05) were submitted to a functional annotation analysis and clustering, based on GO terms, through the Database for Annotation, Visualization and Integrated Discovery (DAVID; v6.8).^21,28^ Data from transcriptomics and proteomics were analyzed separately, and for each group, up- and downregulated gene products were run through DAVID independently. For the functional annotation based on GO terms associated with biological processes, a threshold of five genes per term and an Expression Analysis Systematic Explorer (EASE) score of 0.05 were applied. Functional clustering included GO terms related to cellular components, molecular functions, and biological processes and was performed using an 0.05 minimum EASE score. Classification stringency was set to medium and highest for transcriptomics and proteomics data, respectively.

## Results

### Sequencing analysis overview

**FIG. 1.**
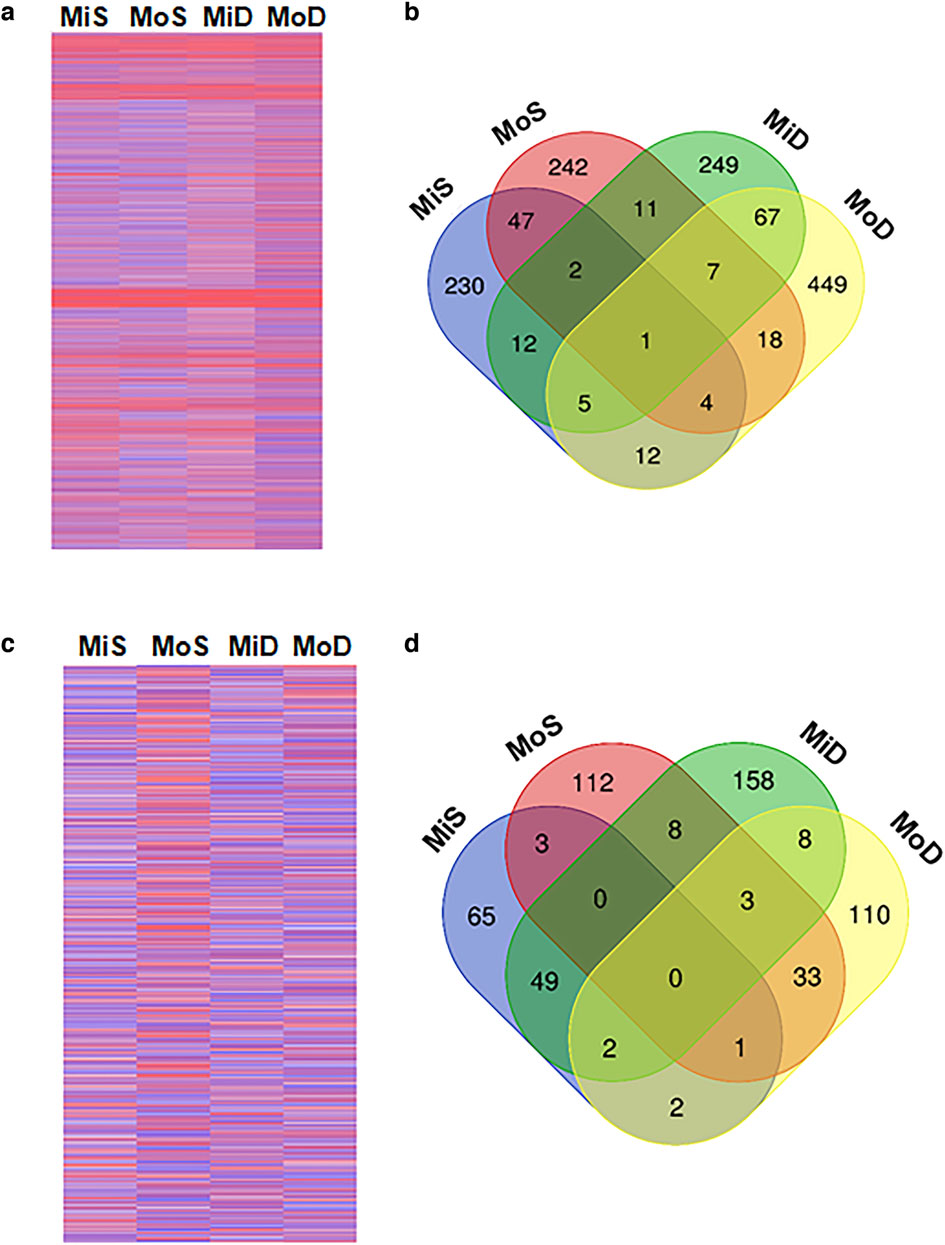
(**A**) Heatmap displaying logFC of all DEGs in the mild single (MiS), moderate single (MoS), mild double (MoD), and moderate double (MoD) conditions. Darker blue represents row minimum whereas darker red represents row maximum. (**B**) Venn diagram displays statistically significant DEGs (*p* < 0.05, *n* = 7). (**C**) Heatmap displaying logFC of all proteins. Darker blue represents row minimum whereas darker red represents row maximum. (**D**) Venn diagram of the significantly up-/downregulated proteins (*p* < 0.05, *n* = 4). DEGs, differentially expressed genes; logFC, log fold change.

A heatmap displays log_2_ fold changes (logFC) of differentially expressed genes (DEGs) for all four injury conditions compared to their respective controls is shown (Fig. 1A). A Venn diagram, including all statistically significant DEGs (*p* < 0.05) for each condition (Fig. 1B), shows that 1356 genes were significantly up- or downregulated in at least one condition. MoD had the most unique DEGs, with 449, whereas MiS had the least, with 230. Eighty DEGs were significantly up- or downregulated in the double conditions and 54 DEGs in the single conditions. One gene, *relaxin3*, was significantly upregulated in every TBI condition, with a logFC ranging from 2.34 to 3.33 across the four conditions compared to controls.

A heatmap of the 4382 proteins observed by protein sequencing shows the up- and downregulated proteins compared to their respective control (Fig. 1C). A Venn diagram showing only the significant (*p* < 0.05) data show that a total of 554 proteins were up- or downregulated in at least one condition (Fig. 1D). MiD had the most unique significant proteins, with 158, whereas MiS had the least.

**FIG. 2.**
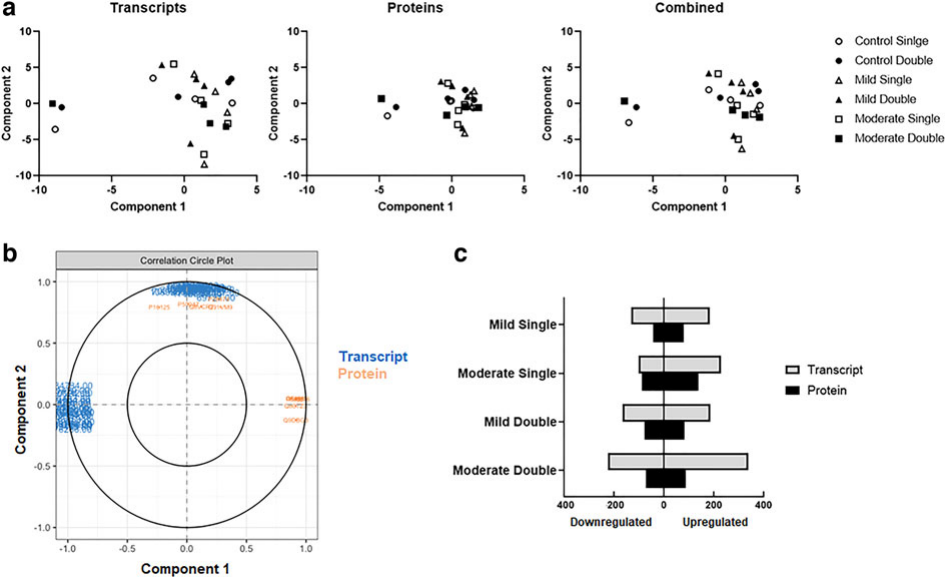
(**A**) Sparse partial least squares (sPLS) individual plots of the transcript and protein data, as well as a combined plot of both the transcript and protein data. (**B**) A correlation circle plot of the transcripts (blue) and proteins (orange). Strongly associated variables are plotted the same direction out from the origin, and the greater the distance from the origin the greater the correlation. The mixOmics R package was used to do sPLS analysis and create the correlation plot. (**C**) The number of up- and downregulated transcripts and proteins for each condition.

**FIG. 3.**
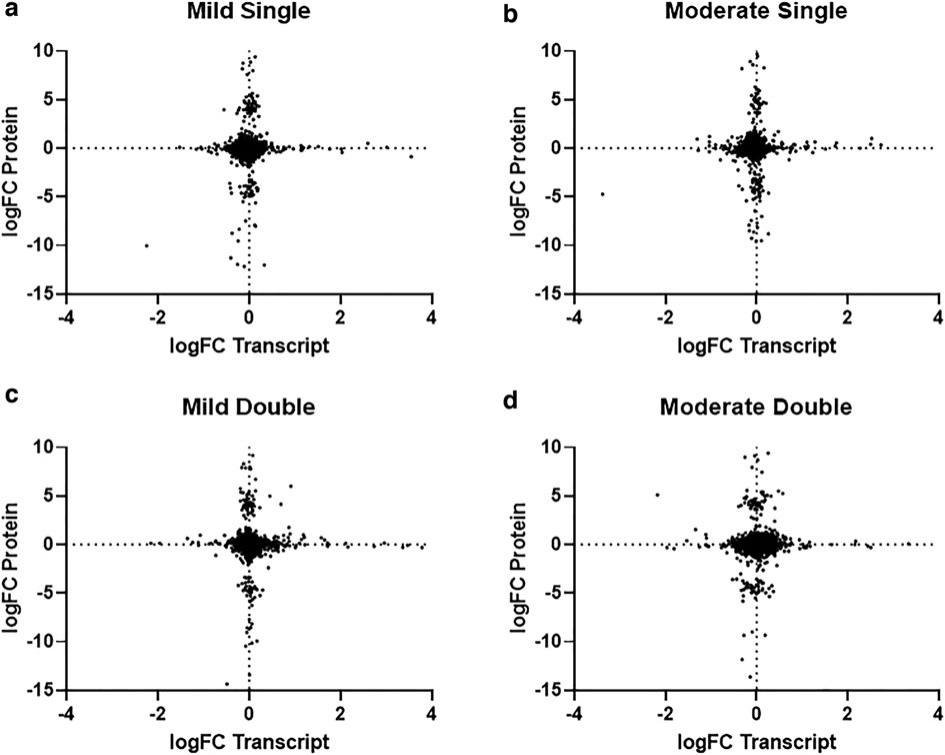
(**A–D**) LogFC of genes were plotted against the logFC of the corresponding proteins for each of the conditions. logFC, log fold change.

Sparse partial least squares regression was performed, and plots representing the effects of conditions across the different platforms are shown (Fig. 2A). These matrices were used to create a correlation circle plot (Fig. 2B), where strongly associated variables were plotted the same distance from the origin, and the further from the origin the more correlated the samples. The total number of significantly up- and downregulated genes and proteins were also plotted (Fig. 2C), and the logFC of the transcriptome and proteome data for corresponding genes in each condition is described in Figure 3. The logFC of significantly up-/downregulated genes pertaining to key biological processes is also shown (Fig. 5 and Supplementary Figs. S1 and S2).

### Neurogenesis

**FIG. 4.**
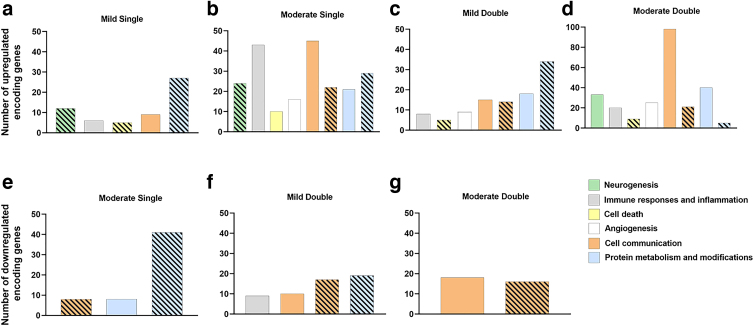
Significantly up- and downregulated gene products were matched with their respective encoding genes, and the biological processes to which they are associated were identified as Gene Ontology terms through DAVID. Graphs represent the number of upregulated (**A–D**) and downregulated (**E–G**) genes encoding transcripts (solid bars) and proteins (striped bars) associated with biological processes relevant to cellular and molecular responses to TBI. DAVID, Database for Annotation, Visualization and Integrated Discovery; TBI, traumatic brain injury.

Of the total upregulated proteins in the MiS condition, 15.2% (12 proteins) were categorized in nine GO terms involved with neurogenesis and neuron development and differentiation, whereas 17.4% (24 proteins) of MoS were associated with 14 similar terms (Fig. 4A,B). For MiS, terms were mainly associated with neuron development and differentiation, regulation of neurogenesis, and development of neuron projections (Supplementary Table S1). Further, functional clustering of all upregulated proteins in MiS included one cluster comprising positive regulation of neuron differentiation, neurogenesis, and cell development, which showed an enrichment score of 2.56—the highest for this group (Table 1). In addition to the above-mentioned terms, after a MoS TBI, upregulated proteins were also categorized in the axon development, dendrite development, and ensheathment of neurons GO terms (Supplementary Table S2). In the MoD group, 9.7% of upregulated transcripts (33 proteins; Fig. 4D) were classified in four neurogenesis-related terms, including neuron fate commitment (Supplementary Table S4). Regarding functional clustering, the terms neurogenesis, positive regulation of neurogenesis, and regulation of neurogenesis were included in one of the eight clusters observed after upregulated transcripts clustering for MoD, with an enrichment score of 1.79 (Table 4). No gene products were associated with any of the above-mentioned GO terms in the MiD group.

**FIG. 5.**
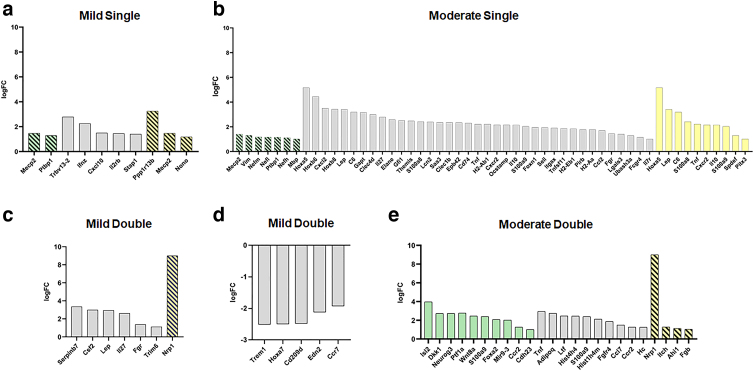
LogFC values of the genes associated with transcripts (solid bars) and proteins (stripped bars) for each biological process were plotted (**A–E**). Biological processes included neurogenesis (green), inflammation/immune response (gray), and cell death (yellow). logFC, log fold change.

**Table 1.** Partial Functional Annotation Clustering Results for Proteins that Had Their Expression Levels Significantly Changed after a Single Mild TBI

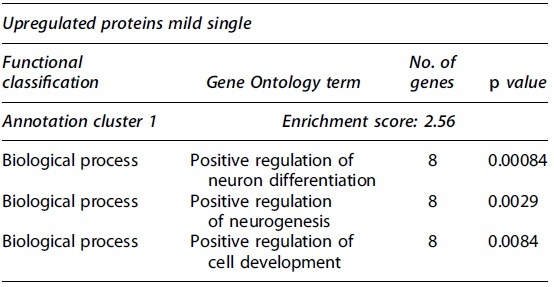

Gene Ontology terms based on biological processes, cellular components, and molecular functions sharing gene members and functions were clustered through DAVID. Clusters considered functionally relevant to molecular responses to TBI are included. The number of encoding genes associated with each term are shown, while *p* values derived from EASE scores demonstrate the gene enrichment in the annotated terms. Full clustering results are available in Supplementary Table S6.

DAVID, Database for Annotation, Visualization and Integrated Discovery; EASE, Expression Analysis Systematic Explorer; TBI, traumatic brain injury.

### Immune responses and inflammation

One biological process was upregulated relating to inflammation in the MiS (cytokine-mediated signaling) and MiD conditions (positive regulation of cytokine production) in the transcriptome level (Supplementary Tables S1 and S3). The MoS group had 45 biological processes and the MoD group revealed five biological processes related to inflammation and immune response in the transcriptome data (Supplementary Tables S2 and S4). For the MoS condition, leukocyte cell-cell adhesion was the term with the most associated genes, including tumor necrosis factor (TNF; logFC 2.22). Other notable biological processes include leukocyte aggregation (16 genes), cellular response to cytokine stimulus (14 genes), and leukocyte migration (13 genes). The MiD condition was the only condition to have downregulated processes regarding inflammation, and eight total downregulated biological processes were found in the transcript data (Fig. 4F). Contrasting with the MoS condition, leukocyte migration had the most downregulated genes (seven) in the MiD condition. Functional annotation clusters including GO terms associated with immune responses and inflammation were observed for upregulated transcripts following MoS (Table 2), MiD (Table 3), and MoD (Table 4) TBI, as well as for downregulated transcripts in response to two mild impacts (Table 3). Most notably, 7 clusters were identified in the MoS group, with enrichment scores ranging from 1.92 to 3.42.

**Table 2.** Partial Functional Annotation Clustering Results for Transcripts that Had Their Expression Levels Significantly Changed after a Single Moderate TBI

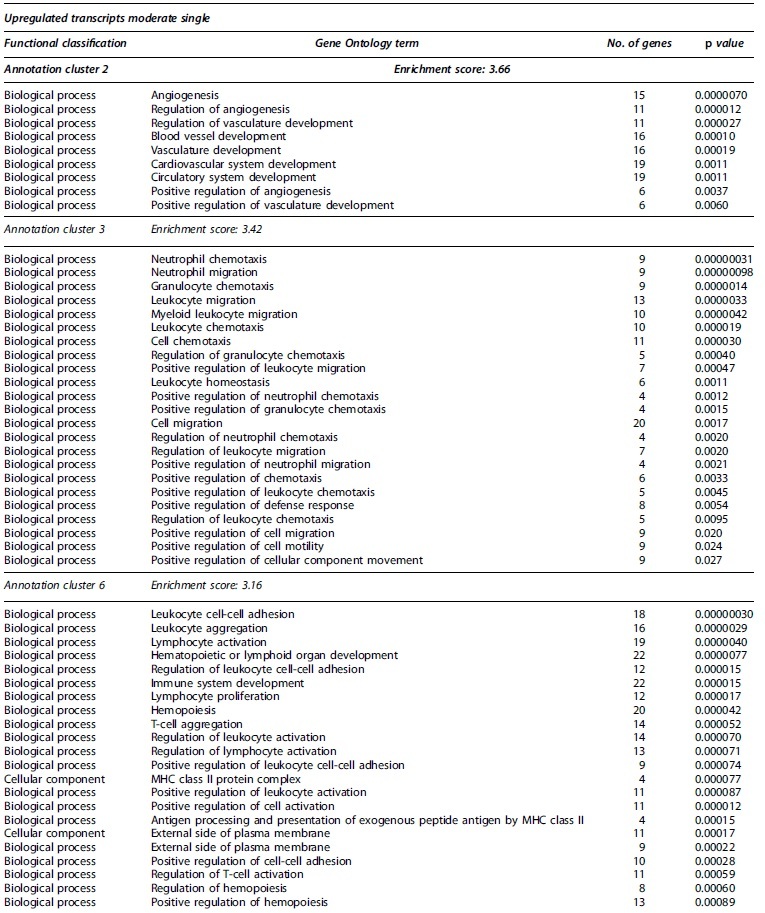 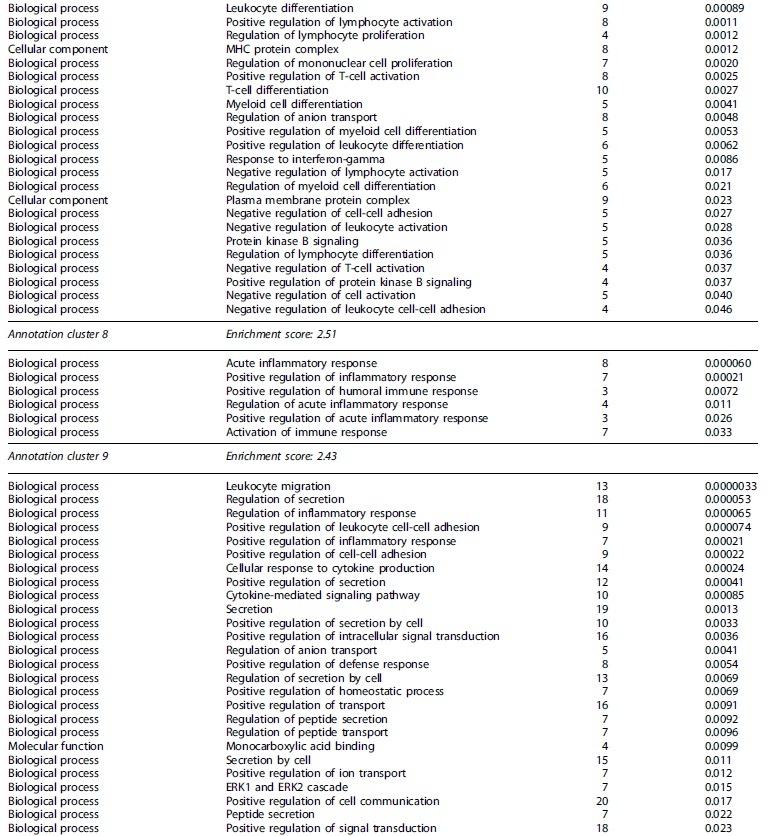 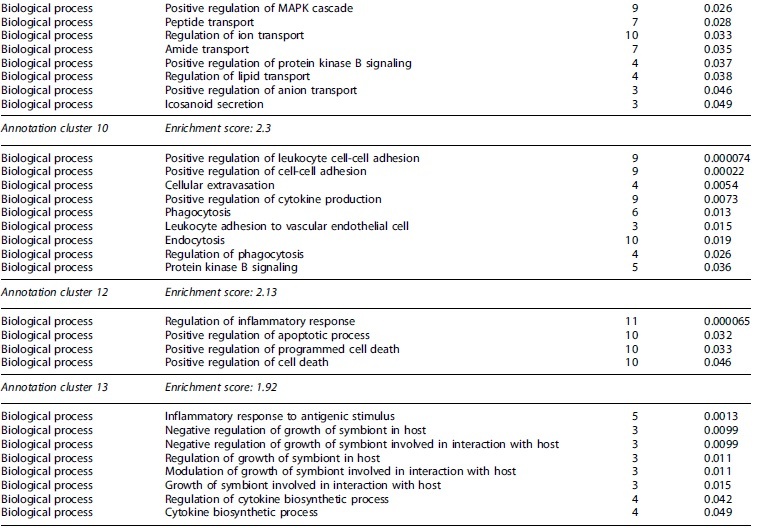

Gene Ontology terms based on biological processes, cellular components, and molecular functions sharing gene members and functions were clustered through DAVID. Clusters considered functionally relevant to molecular responses to TBI are included. The number of encoding genes associated with each term are shown, while *p* values derived from EASE scores demonstrate the gene enrichment in the annotated terms. Full clustering results are available in Supplementary Table S7.

DAVID, Database for Annotation, Visualization and Integrated Discovery; EASE, Expression Analysis Systematic Explorer; ERK1/2, extracellular signal-regulated kinase 1 and 2; MAPK, mitogen-activated protein kinase; MHC, major histocompatibility class; TBI, traumatic brain injury.

**Table 3.** Partial Functional Annotation Clustering Results for Transcripts that Had Their Expression Levels Significantly Changed after Double Mild TBIs

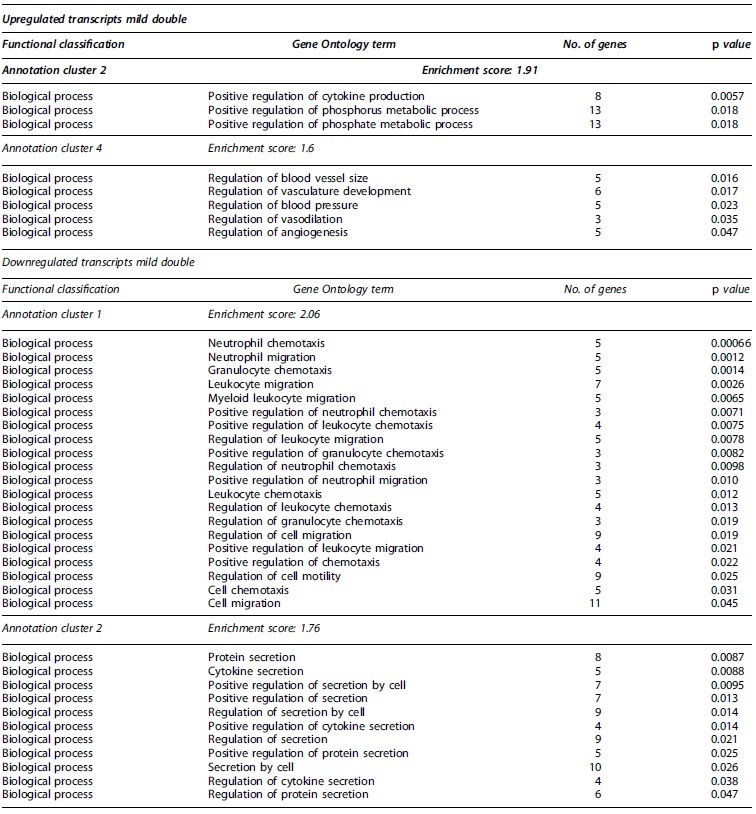

Gene Ontology terms based on biological processes, cellular components, and molecular functions sharing gene members and functions were clustered through DAVID. Clusters considered functionally relevant to molecular responses to TBI are included. The number of encoding genes associated with each term are shown, while *p* values derived from EASE scores demonstrate the gene enrichment in the annotated terms. Full clustering results are available in Supplementary Table S9.

DAVID, Database for Annotation, Visualization and Integrated Discovery; EASE, Expression Analysis Systematic Explorer; TBI, traumatic brain injury.

**Table 4.** Partial Functional Annotation Clustering Results for Transcripts that Had Their Expression Levels Significantly Changed after Double Moderate TBIs

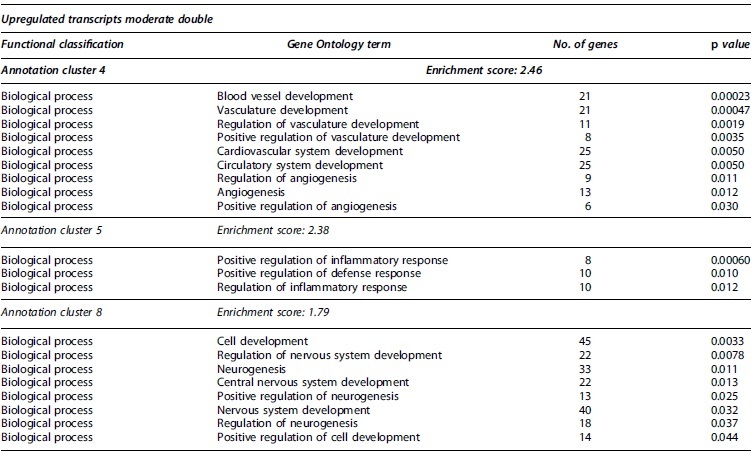

Gene Ontology terms based on biological processes, cellular components, and molecular functions sharing gene members and functions were clustered through DAVID. Clusters considered functionally relevant to molecular responses to TBI are included. The number of encoding genes associated with each term are shown, while *p* values derived from EASE scores demonstrate the gene enrichment in the annotated terms. Full clustering results are available in Supplementary Table S11.

DAVID, Database for Annotation, Visualization and Integrated Discovery; EASE, Expression Analysis Systematic Explorer; TBI, traumatic brain injury.

### Cell death

Functional annotation categorized at least five upregulated gene products in cell-death–related GO terms for each condition. Among the proteins that were significantly upregulated after MiS and MiD TBI, five were associated with regulation of neuron death (Fig. 4A,B), representing 6.3% and 6% of all upregulated proteins in the MiS and MiD groups, respectively. After an MoS injury, 4.4% of upregulated transcripts (10 transcripts) were associated with pro-cell-death stimuli (Fig. 4B), being functionally categorized simultaneously in the positive regulation of cell death, programmed cell death, and apoptotic process GO terms (Supplementary Table S2). For samples obtained after two moderate impacts, 10.5% of upregulated proteins (nine transcripts) were associated with negative regulation of cell death, suggesting the activation of antiapoptotic mechanisms (Supplementary Table S4). Regarding functional clustering, terms associated with cell death and apoptosis were only identified for the MoS group, in the transcriptome level, clustered with an enrichment score of 2.13 and including one term associated with regulation of inflammatory responses in medium stringency settings (Table 2). More results involving other categories can be found in the Supplementary Text.

## Discussion

Persons who previously experienced a TBI have the highest risk of suffering a second injury and developing downstream pathologies.^29,30^ Therefore, we used a closed-head TBI model to study how both injury severity and frequency impacts the cerebral transcriptome and proteome, aiming to identify the biological processes that could be affected. Through functional enrichment analysis, we were able to match significantly altered transcripts and proteins with their respective encoding genes and identify the biological processes to which those genes are functionally associated. Among all the GO terms observed for each group (Supplementary Tables S1–S4), we focused on three main categories relevant to cellular and molecular responses to injury: neurogenesis; immune responses and inflammation; and cell death.

Neural progenitor cell populations enable limited proliferation and differentiation of neural cells in the adult brain in the hippocampal dentate gyrus and the subventricular zone of rodent and human brains.^31^ Upregulated proteins associated with neurogenesis and neuronal development were identified after mild single and moderate single injuries, suggesting that repair-associated mechanisms were functionally activated after a single TBI. Further, the MoS group showed twice the number of significantly upregulated proteins associated with these processes when compared to the MiS condition, indicating that injury severity may impact the extent of activation of neuronal recovery and cellular repopulation mechanisms. Activation of endogenous repair and regeneration processes after brain injury was previously suggested, leading to increased levels of cell proliferation and neurogenesis, and, although limited, it has been associated with spontaneous cognitive improvement in rats submitted to fluid percussion injury.^31–34^ In humans, the presence of proteins associated with neurite outgrowth and synapses was previously reported in microvesicles and exosomes isolated from the cerebrospinal fluid of TBI patients, evidencing the importance of this biological process in the cascade of molecular events triggered by brain injury and suggesting its potential as a TBI biomarker.^35^

In contrast, our observations also suggest that repeated injuries were not capable of functionally inducing neurogenesis, given that no GO terms associated with this process were identified among upregulated proteins in the MiD and MoD groups. This could be a consequence of the development of sustained secondary injury throughout the 8-day interval between the first TBI and euthanasia. Molecular responses to mechanical injuries include pathological processes, such as ischemia, excitotoxicity, proapoptotic signaling, oxidative stress, and inflammation, which create a hostile microenvironment that can impair endogenous neurogenesis.^19,36,37^

In addition to neurogenesis, vasculogenesis and angiogenesis are also important mediators of functional recovery after experimental TBI. A better understanding of how these processes are activated after injury, and their crosstalk, can lead to the identification of therapeutic targets.^36^ Our functional analysis showed that transcripts associated with angiogenesis and blood vessel development were significantly upregulated in the MoS, MiD, and MoD groups (Supplementary Text). Vascular damage is a major consequence of TBI and it plays a key role in the development of secondary injury through edema, blood flow impairments, and blood–brain barrier disruption, evidencing the importance of addressing vascular dysfunctions in the context of TBI recovery.^23,38^ Although the mechanisms involved in vascular repair are poorly understood, it has been suggested that the process is initiated between 2 and 3 weeks after TBI.^38^ In this context, our results suggest that repair-associated genes are transcribed shortly after injury, whereas functional alterations in protein level are achieved beyond the time bounds of our experiments.

Inflammation, an innate immune response, is a well-characterized long-term response of TBI.^39–41^ After TBI, the cerebral tissue undergoes pro- and anti-inflammatory cytokine production, microglial activation, and immune cell recruitment.^40,41^ Neuroinflammation can have damaging or beneficial effects on brain tissue.^42^ Current research aims to tease out the neurotropic and neurotoxic effects to develop anti-inflammatory treatments.^43^ Although we reported an increase in inflammation processes, further research is needed to determine whether the specific processes we report are beneficial or detrimental to the cerebral tissue. In every condition, we found significantly upregulated genes associated with each of these immune responses.

Accordingly, previous GO-based functional analysis of differentially expressed transcripts in mice hippocampus after controlled cortical impact injury showed the association of upregulated transcripts with five GO terms associated with the regulation of immune responses, including inflammatory response and regulation of cytokine production.^44^ The MoS condition had 45 upregulated processes dealing with inflammation, which was the most of all conditions. Previous reports have shown that the severity of the injury dictates the recruitment of other immune cells, explaining the dramatic increase in the number of immune cell migrations we report in the MoS condition.^45^ Previous reports have also found that closed-head mouse models undergoing repeated injuries, spaced 3 days apart, elicited a greater inflammatory transcriptome response than those spaced 20 days apart.^4^ Although we saw a large response in the MoS condition, we did not observe the same response in the MoD. We speculate that transcripts in the double conditions did not have as robust of a response as the MoS condition because the immune system, specifically the microglial, was already primed from the previous injury.^46^

Inflammation can also lead to cell death, or apoptosis.^47^ We found that proteins or transcripts involved in cell death processes significantly upregulated in all conditions. TNF, a proinflammatory cytokine that can induce inflammation, is a major contributor to apoptotic cell death. *TNF* was upregulated to some extent in every condition post-TBI and significantly upregulated in the MoS and both double TBI conditions. Past studies have demonstrated that mice lacking the proteins TNFα and its cell death receptor, Fas, showed decreased brain damage compared to wild-type mice.^48^ Our findings of significantly increased *TNF* in the cortical transcriptome after moderate TBI is consistent with previous studies regarding cell death and tissue damage.^49^

To gain a comprehensive view of the damaged tissue post-injury, both the transcriptome and proteome were analyzed. The transcriptome and proteome are not isolated entities, and both should be taken into account when interpreting results; however the relationship between the proteome and transcriptome is not linear.^22,50^ It should be noted that protein expression is more conserved than transcription expression, and DEGs are more likely to correlate with protein changes.^50,51^ We acknowledge that not all transcriptional changes represent changes in the proteome, but understand that DEGs will provide a more global approach to understand the pathophysiology after repeated TBIs.

## Conclusion

Using our established closed-head TBI model, we analyzed the transcriptome and proteome response after repeated injuries of different magnitudes. After a single injury, transcriptional analysis showed that neurogenesis pathways were upregulated. Neuroinflammation was present in all conditions and, pointedly, in the moderate single condition. Apoptosis was upregulated after moderate and repeated injuries. Our results emphasize the significant differences found in proteomic and transcriptomic changes in single versus double injuries. Further, cortical omics analysis offers important insights for future studies aiming to deepen the current knowledge on the development of secondary injuries after brain trauma.

## Supplementary Material

Supplemental data

Supplemental data

Supplemental data

Supplemental data

Supplemental data

Supplemental data

Supplemental data

Supplemental data

Supplemental data

Supplemental data

Supplemental data

Supplemental data

Supplemental data

Supplemental data

Supplemental data

## Abbreviations Used

DAVID: Database for Annotation, Visualization and Integrated Discovery
DEGs: differentially expressed genes
EASE: Expression Analysis Systematic Explorer
GO: Gene Ontology
logFC: log_2_ fold changes
MiD: mild double TBI
MiS: mild single TBI
MoD: moderate double TBI
MoS: moderate single TBI
RNA-seq: RNA-sequencing
TBI: traumatic brain injury
TNF: tumor necrosis factor
