# Supplementary material for: Functional Analysis of the Cortical Transcriptome and Proteome Reveal Neurogenesis, Inflammation, and Cell Death after Repeated Traumatic Brain Injury In vivo
Source: Neurotrauma Rep. 2022 Jun 13;3(1):224–39. doi: 10.1089/neur.2021.0059 (PMC9279125; doi:10.1089/neur.2021.0059)

**Supplemental Figure 1:** The logFC values of the genes associated with transcripts (solid bars) and proteins (stripped bars) for each biological process were plotted for the mild single and moderate single conditions (a-c). The biological processes included angiogenesis (white), cell communication (orange) and protein metabolism (blue).


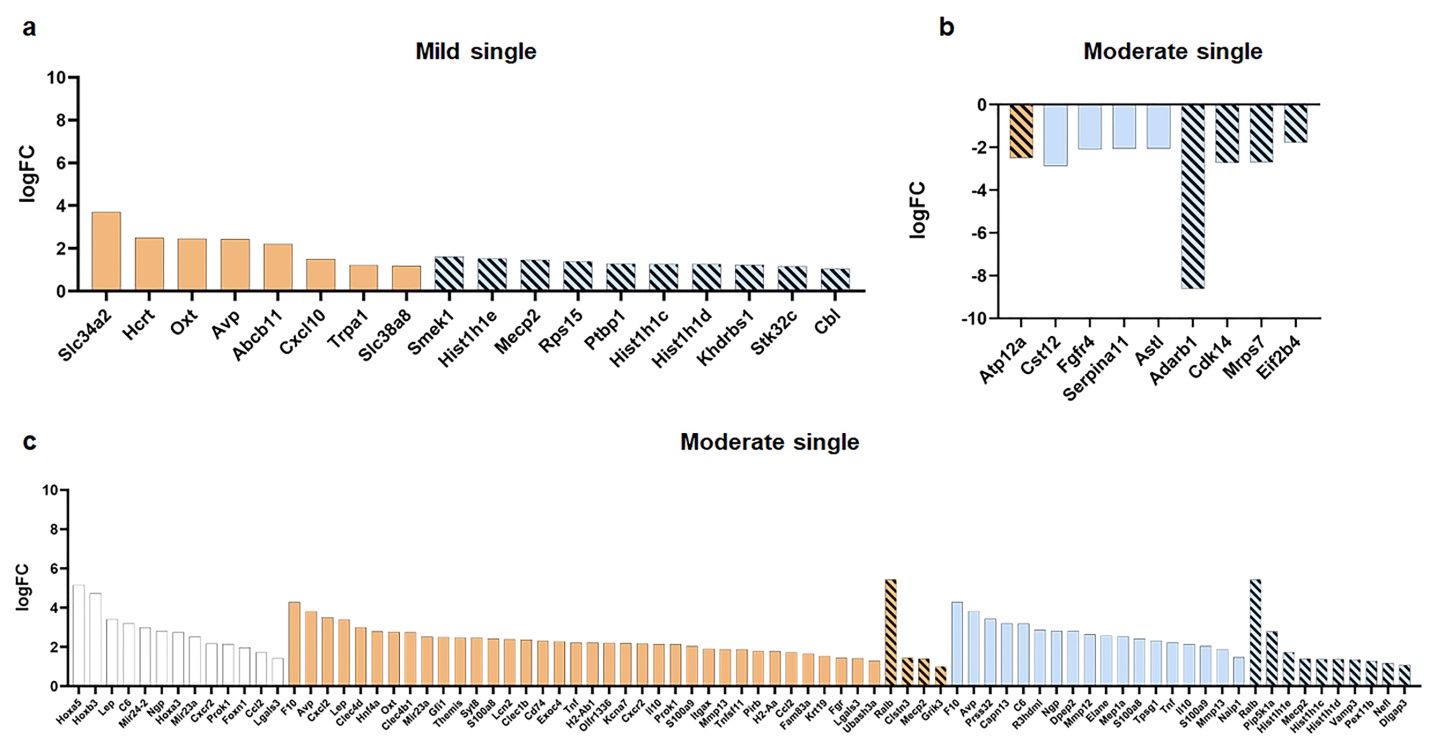

Supplement: Supplemental data [file Suppl_FigS1.docx]
