# Supplementary material for: Functional Analysis of the Cortical Transcriptome and Proteome Reveal Neurogenesis, Inflammation, and Cell Death after Repeated Traumatic Brain Injury In vivo
Source: Neurotrauma Rep. 2022 Jun 13;3(1):224–39. doi: 10.1089/neur.2021.0059 (PMC9279125; doi:10.1089/neur.2021.0059)
Supplement: Supplemental data [file Suppl_TableS1.docx]

**Supplemental table 1:** Functional annotation of transcripts and proteins which had their expression levels significantly altered following a single mild traumatic brain injury. Data shows the number of encoding genes associated with Gene Ontology terms representing biological processes. The p-values are derived from EASE-scores and demonstrate the gene enrichment in the annotated terms.

| **UPREGULATED TRANSCRIPTS SINGLE MILD** | | |
| --- | --- | --- |
| **Biological process** | **Number of genes** | **P-value** |
| Anion transport | 7 | 0.02 |
| Cytokine-mediated signaling pathway | 6 | 0.03 |
| Positive regulation of ion transport | 6 | 0.01 |
|  |  |  |
| **UPREGULATED PROTEINS SINGLE MILD** | | |
| **Biological process** | **Number of genes** | **P-value** |
| Cellular protein metabolic process | 27 | 0.02 |
| Neuron differentiation | 12 | 0.02 |
| Regulation of neurogenesis | 10 | 0.005 |
| Neuron projection development | 10 | 0.01 |
| Neuron development | 10 | 0.03 |
| Regulation of neuron differentiation | 9 | 0.005 |
| Peptide metabolic process | 9 | 0.02 |
| Positive regulation of neuron differentiation | 8 | 0.001 |
| Positive regulation of neurogenesis | 8 | 0.003 |
| Regulation of neuron projection development | 7 | 0.02 |
| Positive regulation of neuron projection development | 6 | 0.01 |
| Regulation of neuron death | 5 | 0.04 |
| Protein methylation | 5 | 0.004 |
