# Supplementary material for: Functional Analysis of the Cortical Transcriptome and Proteome Reveal Neurogenesis, Inflammation, and Cell Death after Repeated Traumatic Brain Injury In vivo
Source: Neurotrauma Rep. 2022 Jun 13;3(1):224–39. doi: 10.1089/neur.2021.0059 (PMC9279125; doi:10.1089/neur.2021.0059)
Supplement: Supplemental data [file Suppl_TableS2.docx]

**Supplemental table 2:** Functional annotation of transcripts and proteins which had their expression levels significantly altered following a single moderate traumatic brain injury. Data shows the number of encoding genes associated with Gene Ontology terms representing biological processes. The p-values are derived from EASE-scores and demonstrate the gene enrichment in the annotated terms.

| **UPREGULATED TRANSCRIPTS SINGLE MODERATE** | | |
| --- | --- | --- |
| **Biological process** | **Number of genes** | **P-value** |
| Cell surface receptor signaling pathway | 27 | 0.02 |
| Positive regulation of cell communication | 20 | 0.02 |
| Secretion | 19 | 0.001 |
| Leukocyte cell-cell adhesion | 18 | 0.0000003 |
| Positive regulation of signal transduction | 18 | 0.02 |
| Blood vessel development | 16 | 0.0001 |
| Vasculature development | 16 | 0.0002 |
| Leukocyte aggregation | 16 | 0.000003 |
| Positive regulation of intracellular signal transduction | 16 | 0.004 |
| Positive regulation of transport | 16 | 0.01 |
| Angiogenesis | 15 | 0.00001 |
| Secretion by cell | 15 | 0.01 |
| T cell aggregation | 14 | 0.0001 |
| Regulation of leukocyte activation | 14 | 0.0001 |
| Cellular response to cytokine stimulus | 14 | 0.0002 |
| Leukocyte migration | 13 | 0.000003 |
| Regulation of lymphocyte activation | 13 | 0.0001 |
| Leukocyte differentiation | 13 | 0.001 |
| Regulation of secretion by cell | 13 | 0.01 |
| Regulation of leukocyte cell-cell adhesion | 12 | 0.00002 |
| Lymphocyte proliferation | 12 | 0.00002 |
| Positive regulation of secretion | 12 | 0.0004 |
| Regulation of angiogenesis | 11 | 0.00001 |
| Regulation of vasculature development | 11 | 0.00003 |
| Regulation of inflammatory response | 11 | 0.0001 |
| Positive regulation of leukocyte activation | 11 | 0.0001 |
| Positive regulation of apoptotic process | 10 | 0.03 |
| Positive regulation of programmed cell death | 10 | 0.03 |
| Positive regulation of cell death | 10 | 0.05 |
| Myeloid leukocyte migration | 10 | 0.000004 |
| Leukocyte chemotaxis | 10 | 0.00002 |
| Myeloid cell differentiation | 10 | 0.003 |
| Positive regulation of secretion by cell | 10 | 0.003 |
| Regulation of ion transport | 10 | 0.03 |
| Neutrophil chemotaxis | 9 | 0.0000003 |
| Neutrophil migration | 9 | 0.000001 |
| Granulocyte chemotaxis | 9 | 0.000001 |
| Positive regulation of leukocyte cell-cell adhesion | 9 | 0.0001 |
| Positive regulation of lymphocyte activation | 9 | 0.001 |
| Positive regulation of cytokine production | 9 | 0.01 |
| Acute inflammatory response | 8 | 0.0001 |
| Regulation of lymphocyte proliferation | 8 | 0.001 |
| Regulation of mononuclear cell proliferation | 8 | 0.001 |
| T cell differentiation | 8 | 0.003 |
| Regulation of leukocyte differentiation | 8 | 0.005 |
| Positive regulation of inflammatory response | 7 | 0.0002 |
| Positive regulation of leukocyte migration | 7 | 0.0005 |
| B cell mediated immunity | 7 | 0.001 |
| Positive regulation of T cell activation | 7 | 0.002 |
| Regulation of leukocyte migration | 7 | 0.002 |
| Activation of immune response | 7 | 0.03 |
| Regulation of peptide secretion | 7 | 0.01 |
| Regulation of peptide transport | 7 | 0.01 |
| Positive regulation of ion transport | 7 | 0.01 |
| Peptide secretion | 7 | 0.02 |
| Peptide transport | 7 | 0.03 |
| Amide transport | 7 | 0.04 |
| Positive regulation of angiogenesis | 6 | 0.004 |
| Positive regulation of vasculature development | 6 | 0.01 |
| Leukocyte homeostasis | 6 | 0.001 |
| Response to interleukin-1 | 6 | 0.002 |
| Positive regulation of leukocyte differentiation | 6 | 0.01 |
| Negative regulation of secretion | 6 | 0.04 |
| Regulation of granulocyte chemotaxis | 5 | 0.0004 |
| Positive regulation of leukocyte chemotaxis | 5 | 0.005 |
| Positive regulation of myeloid cell differentiation | 5 | 0.01 |
| Response to interferon-gamma | 5 | 0.01 |
| Regulation of leukocyte chemotaxis | 5 | 0.01 |
| Negative regulation of lymphocyte activation | 5 | 0.02 |
| Regulation of adaptive immune response based on somatic recombination of immune receptors built from immunoglobulin superfamily domains | 5 | 0.03 |
| Negative regulation of leukocyte activation | 5 | 0.03 |
| Myeloid cell homeostasis | 5 | 0.03 |
| Regulation of lymphocyte differentiation | 5 | 0.04 |
| Regulation of anion transport | 5 | 0.004 |
| Negative regulation of cell-cell adhesion | 5 | 0.03 |
|  |  |  |
| **DOWNREGULATED TRANSCRIPTS SINGLE MODERATE** | | |
| **Biological process** | **Number of genes** | **P-value** |
| Regulation of proteolysis | 8 | 0.003 |
| Positive regulation of proteolysis | 5 | 0.01 |
|  |  |  |
| **UPREGULATED PROTEINS SINGLE MODERATE** | | |
| **Biological process** | **Number of genes** | **P-value** |
| Neurogenesis | 24 | 0.001 |
| Neuron differentiation | 23 | 0.0002 |
| Neuron projection development | 22 | 0.000001 |
| Neuron development | 22 | 0.00002 |
| Protein complex assembly | 19 | 0.003 |
| Neuron projection morphogenesis | 14 | 0.0002 |
| Regulation of neurogenesis | 14 | 0.005 |
| Trans-synaptic signaling | 14 | 0.0002 |
| Synaptic signaling | 14 | 0.0002 |
| Regulation of neuron projection development | 13 | 0.0002 |
| Regulation of neuron differentiation | 13 | 0.003 |
| Regulation of vesicle-mediated transport | 12 | 0.0003 |
| Cell morphogenesis involved in neuron differentiation | 11 | 0.004 |
| Modulation of synaptic transmission | 11 | 0.0001 |
| Protein localization to membrane | 11 | 0.001 |
| Cellular protein complex assembly | 10 | 0.01 |
| Axon development | 9 | 0.01 |
| Positive regulation of neurogenesis | 9 | 0.02 |
| Positive regulation of neuron projection development | 7 | 0.02 |
| Positive regulation of synaptic transmission | 7 | 0.001 |
| Dendrite development | 6 | 0.03 |
| Ensheathment of neurons | 5 | 0.01 |
| Negative regulation of protein polymerization | 5 | 0.001 |
| Negative regulation of protein complex disassembly | 5 | 0.001 |
| Regulation of protein depolymerization | 5 | 0.002 |
| Negative regulation of protein complex assembly | 5 | 0.01 |
| Protein methylation | 5 | 0.03 |
|  |  |  |
| **DOWNREGULATED PROTEINS SINGLE MODERATE** | | |
| **Biological process** | **Number of genes** | **P-value** |
| Cellular protein metabolic process | 32 | 0.03 |
| Regulation of protein metabolic process | 20 | 0.03 |
| Proteolysis | 17 | 0.004 |
| Protein catabolic process | 13 | 0.0004 |
| Proteolysis involved in cellular protein catabolic process | 12 | 0.0002 |
| Cellular protein catabolic process | 12 | 0.0004 |
| Peptide metabolic process | 10 | 0.03 |
| Cation transmembrane transport | 8 | 0.02 |
| Inorganic ion transmembrane transport | 8 | 0.02 |
| Regulation of ion transmembrane transporter activity | 5 | 0.02 |
| Regulation of transmembrane transporter activity | 5 | 0.02 |
| Regulation of transporter activity | 5 | 0.02 |
| Regulation of cation transmembrane transport | 5 | 0.03 |
