# Supplementary material for: Functional Analysis of the Cortical Transcriptome and Proteome Reveal Neurogenesis, Inflammation, and Cell Death after Repeated Traumatic Brain Injury In vivo
Source: Neurotrauma Rep. 2022 Jun 13;3(1):224–39. doi: 10.1089/neur.2021.0059 (PMC9279125; doi:10.1089/neur.2021.0059)
Supplement: Supplemental data [file Suppl_TableS4.docx]

**Supplemental table 4:** Functional annotation of transcripts and proteins which had their expression levels significantly altered following double moderate traumatic brain injuries. Data shows the number of encoding genes associated with Gene Ontology terms representing biological processes. The p-values are derived from EASE-scores and demonstrate the gene enrichment in the annotated terms.

| **UPREGULATED TRANSCRIPTS DOUBLE MODERATE** | | |
| --- | --- | --- |
| **Biological process** | **Number of genes** | **P-value** |
| Cell surface receptor signaling pathway | 49 | 0.0003 |
| Regulation of signal transduction | 42 | 0.05 |
| Neurogenesis | 33 | 0.01 |
| Ion transport | 32 | 0.001 |
| Regulation of protein modification process | 30 | 0.04 |
| Circulatory system development | 25 | 0.01 |
| Cardiovascular system development | 25 | 0.01 |
| Blood vessel development | 21 | 0.0002 |
| Vasculature development | 21 | 0.0005 |
| Negative regulation of protein metabolic process | 21 | 0.04 |
| Regulation of neurogenesis | 18 | 0.04 |
| Regulation of protein transport | 17 | 0.05 |
| Anion transport | 16 | 0.0004 |
| Regulation of proteolysis | 15 | 0.03 |
| Positive regulation of neurogenesis | 13 | 0.03 |
| Angiogenesis | 13 | 0.01 |
| Regulation of vasculature development | 11 | 0.002 |
| Organic anion transport | 11 | 0.01 |
| Regulation of inflammatory response | 10 | 0.01 |
| Regulation of angiogenesis | 9 | 0.01 |
| Lipid transport | 9 | 0.04 |
| Positive regulation of vasculature development | 8 | 0.004 |
| Positive regulation of inflammatory response | 8 | 0.001 |
| Negative regulation of cytokine production | 8 | 0.02 |
| Positive regulation of angiogenesis | 6 | 0.03 |
| Neuron fate commitment | 5 | 0.02 |
| Regulation of chemokine production | 5 | 0.02 |
| Negative regulation of myeloid cell differentiation | 5 | 0.04 |
|  |  |  |
| **DOWNREGULATED TRANSCRIPTS DOUBLE MODERATE** | | |
| **Biological process** | **Number of genes** | **P-value** |
| Ion transport | 16 | 0.03 |
| Transmembrane transport | 15 | 0.006 |
| Ion transmembrane transport | 10 | 0.04 |
|  |  |  |
| **UPREGULATED PROTEINS DOUBLE MODERATE** | | |
| **Biological process** | **Number of genes** | **P-value** |
| Protein transport | 15 | 0.006 |
| Intracellular protein transport | 10 | 0.008 |
| Negative regulation of cell death | 9 | 0.05 |
| Positive regulation of transport | 9 | 0.04 |
| Regulation of protein transport | 8 | 0.03 |
| Regulation of establishment of protein localization | 8 | 0.05 |
| Regulation of vesicle-mediated transport | 7 | 0.008 |
| Trans-synaptic signaling | 7 | 0.02 |
| Synaptic signaling | 7 | 0.02 |
| Positive regulation of protein transport | 6 | 0.04 |
| Positive regulation of cellular protein localization | 5 | 0.05 |
| Protein maturation | 5 | 0.02 |
|  |  |  |
| **DOWNREGULATED PROTEINS DOUBLE MODERATE** | | |
| **Biological process** | **Number of genes** | **P-value** |
| Protein transport | 16 | 0.002 |
| Intracellular protein transport | 12 | 0.0004 |
