# Supplementary material for: Functional Analysis of the Cortical Transcriptome and Proteome Reveal Neurogenesis, Inflammation, and Cell Death after Repeated Traumatic Brain Injury In vivo
Source: Neurotrauma Rep. 2022 Jun 13;3(1):224–39. doi: 10.1089/neur.2021.0059 (PMC9279125; doi:10.1089/neur.2021.0059)
Supplement: Supplemental data [file Suppl_TableS6.docx]

**Supplemental table 6:** Functional annotation clustering results for proteins which had their expression levels significantly changed after a single mild traumatic brain injury. Gene Ontology terms based on biological processes, cellular components, and molecular functions sharing gene members and functions were clustered through DAVID. Data shows the number of encoding genes associated with each term, while p-values derived from EASE-scores demonstrate the gene enrichment in the annotated terms.

| UPREGULATED PROTEINS SINGLE MILD | | | |
| --- | --- | --- | --- |
| Functional classification | Gene Ontology Term | Number of genes | **P-value** |
| Annotation cluster 1 | Enrichment score: 2.56 | | |
| Biological process | Positive regulation of neuron differentiation | 8 | 0.00084 |
| Biological process | Positive regulation of neurogenesis | 8 | 0.0029 |
| Biological process | Positive regulation of cell development | 8 | 0.0084 |
| **Annotation cluster 2** | **Enrichment score: 2.31** | | |
| Biological process | Histone metilation | 5 | 0.0016 |
| Biological process | Protein methylation | 5 | 0.0035 |
| Biological process | Macromolecule methylation | 5 | 0.022 |
| **Annotation cluster 3** | **Enrichment score: 1.97** | | |
| Biological process | Translation | 9 | 0.0065 |
| Biological process | Peptide biosynthetic process | 9 | 0.0077 |
| Biological process | Amide biosynthetic process | 9 | 0.013 |
| Biological process | Peptide metabolic process | 9 | 0.020 |
| **Annotation cluster 4** | **Enrichment score: 1.66** | | |
| Biological process | Negative regulation of RNA metabolic process | 12 | 0.010 |
| Biological process | Negative regulation of nucleobase-containing compound metabolic process | 12 | 0.021 |
| Biological process | Negative regulation of gene expression | 12 | 0.050 |
| **Annotation cluster 5** | **Enrichment score: 1.37** | |  |
| Molecular function | Purine ribonucleoside binding | 14 | 0.037 |
| Molecular function | Purine ribonucleotide binding | 14 | 0.043 |
| Molecular function | Purine nucleotide binding | 14 | 0.044 |
| Molecular function | Ribonucleotide binding | 14 | 0.045 |
|  |  |  |  |
| **DOWNREGULATED PROTEINS SINGLE MILD** | | | |
| **Functional classification** | **Gene Ontology Term** | **Number of genes** | **P-value** |
| **Annotation cluster 1** | **Enrichment score: 2.34** | | |
| Biological process | Cerebellum development | 4 | 0.0028 |
| Biological process | Metencephalon development | 4 | 0.0037 |
| Biological process | Hindbrain development | 4 | 0.0093 |
| **Annotation cluster 2** | **Enrichment score: 1.96** | | |
| Molecular function | Gated channel activity | 5 | 0.0058 |
| Molecular function | Ion channel activity | 5 | 0.014 |
| Molecular function | Substrate-specific channel activity | 5 | 0.016 |
