# Supplementary material for: Functional Analysis of the Cortical Transcriptome and Proteome Reveal Neurogenesis, Inflammation, and Cell Death after Repeated Traumatic Brain Injury In vivo
Source: Neurotrauma Rep. 2022 Jun 13;3(1):224–39. doi: 10.1089/neur.2021.0059 (PMC9279125; doi:10.1089/neur.2021.0059)
Supplement: Supplemental data [file Suppl_TableS3.docx]

**Supplemental table 3:** Functional annotation of transcripts and proteins which had their expression levels significantly altered following double mild traumatic brain injuries. Data shows the number of encoding genes associated with Gene Ontology terms representing biological processes. The p-values are derived from EASE-scores and demonstrate the gene enrichment in the annotated terms.

| **UPREGULATED TRANSCRIPTS DOUBLE MILD** | | |
| --- | --- | --- |
| **Biological process** | **Number of genes** | **P-value** |
| Protein complex assembly | 14 | 0.03 |
| Positive regulation of transport | 13 | 0.01 |
| Positive regulation of cytokine production | 8 | 0.006 |
| Positive regulation of secretion | 7 | 0.04 |
| Regulation of vesicle-mediated transport | 7 | 0.05 |
| Regulation of vasculature development | 6 | 0.02 |
| Regulation of blood vessel size | 5 | 0.02 |
| Regulation of blood pressure | 5 | 0.02 |
| Regulation of angiogenesis | 5 | 0.05 |
| Protein processing | 5 | 0.04 |
|  |  |  |
| **DOWNREGULATED TRANSCRIPTS DOUBLE MILD** | | |
| **Biological process** | **Number of genes** | **P-value** |
| Secretion by cell | 10 | 0.03 |
| Regulation of secretion by cell | 9 | 0.01 |
| Regulation of secretion | 9 | 0.02 |
| Protein secretion | 8 | 0.009 |
| Leukocyte migration | 7 | 0.003 |
| Positive regulation of secretion by cell | 7 | 0.01 |
| Positive regulation of secretion | 7 | 0.01 |
| Regulation of protein secretion | 6 | 0.05 |
| Neutrophil chemotaxis | 5 | 0.0007 |
| Neutrophil migration | 5 | 0.001 |
| Granulocyte chemotaxis | 5 | 0.001 |
| Myeloid leukocyte migration | 5 | 0.007 |
| Regulation of leukocyte migration | 5 | 0.008 |
| Cytokine secretion | 5 | 0.009 |
| Leukocyte chemotaxis | 5 | 0.01 |
| Positive regulation of protein secretion | 5 | 0.03 |
|  |  |  |
| **UPREGULATED PROTEINS DOUBLE MILD** | | |
| **Biological process** | **Number of genes** | **P-value** |
| Cellular protein metabolic process | 31 | 0.003 |
| Cellular protein modification process | 23 | 0.02 |
| Protein modification process | 23 | 0.02 |
| Protein transport | 14 | 0.02 |
| Proteolysis | 14 | 0.01 |
| Proteolysis involved in cellular protein catabolic process | 9 | 0.004 |
| Cellular protein catabolic process | 9 | 0.005 |
| Protein catabolic process | 9 | 0.01 |
| Regulation of neuron death | 5 | 0.04 |
|  |  |  |
| **DOWNREGULATED PROTEINS DOUBLE MILD** | | |
| **Biological process** | **Number of genes** | **P-value** |
| Protein transport | 15 | 0.005 |
| Protein complex assembly | 11 | 0.02 |
| Intracellular protein transport | 10 | 0.007 |
| Single-organism intracellular transport | 8 | 0.007 |
| Protein localization to organelle | 8 | 0.03 |
| Protein targeting | 8 | 0.008 |
| Establishment of protein localization to organelle | 7 | 0.02 |
| Cellular protein complex assembly | 6 | 0.05 |
| Protein import | 5 | 0.04 |
