# Supplementary material for: Functional Analysis of the Cortical Transcriptome and Proteome Reveal Neurogenesis, Inflammation, and Cell Death after Repeated Traumatic Brain Injury In vivo
Source: Neurotrauma Rep. 2022 Jun 13;3(1):224–39. doi: 10.1089/neur.2021.0059 (PMC9279125; doi:10.1089/neur.2021.0059)
Supplement: Supplemental data [file Suppl_TableS7.docx]

**Supplemental table 7:** Functional annotation clustering results for transcripts which had their expression levels significantly changed after a single moderate traumatic brain injury. Gene Ontology terms based on biological processes, cellular components, and molecular functions sharing gene members and functions were clustered through DAVID. Data shows the number of encoding genes associated with each term, while p-values derived from EASE-scores demonstrate the gene enrichment in the annotated terms.

| UPREGULATED TRANSCRIPTS SINGLE MODERATE | | | |
| --- | --- | --- | --- |
| Functional classification | Gene Ontology Term | Number of genes | **P-value** |
| Annotation cluster 1 | Enrichment score: 3.91 | | |
| Biological process | Anterior/posterior pattern specification | 12 | 0.00000084 |
| Biological process | Embryonic skeletal system develeopment | 10 | 0.0000011 |
| Biological process | Embryonic skeletal system morphogenesis | 9 | 0.0000013 |
| Biological process | Regionalization | 13 | 0.000015 |
| Biological process | Embryonic organ development | 15 | 0.000023 |
| Biological process | Skeletal system development | 15 | 0.000026 |
| Biological process | Organ morphogenesis | 22 | 0.000034 |
| Biological process | Embryonic organ morphogenesis | 12 | 0.000034 |
| Biological process | Skeletal system morphogenesis | 10 | 0.0000087 |
| Biological process | Embryo development ending in birth or egg hatching | 17 | 0.00015 |
| Molecular function | Sequence-specific DNA binding | 20 | 0.00019 |
| Biological process | Embryonic morphogenesis | 14 | 0.0013 |
| Biological process | Cartilage development | 7 | 0.0039 |
| Biological process | Transcription, DNA-templated | 35 | 0.016 |
| Biological process | Regulation of transcription, DNA-templated | 36 | 0.037 |
| Biological process | Regulation of RNA biosynthetic process | 36 | 0.038 |
| **Annotation cluster 2** | **Enrichment score: 3.66** | | |
| Biological process | Angiogenesis | 15 | 0.0000070 |
| Biological process | Regulation of angiogenesis | 11 | 0.000012 |
| Biological process | Regulation of vasculature development | 11 | 0.000027 |
| Biological process | Blood vessel development | 16 | 0.00010 |
| Biological process | Vasculature development | 16 | 0.00019 |
| Biological process | Cardiovascular system development | 19 | 0.0011 |
| Biological process | Circulatory system development | 19 | 0.0011 |
| Biological process | Positive regulation of angiogenesis | 6 | 0.0037 |
| Biological process | Positive regulation of vasculature development | 6 | 0.0060 |
| **Annotation cluster 3** | **Enrichment score: 3.42** | | |
| Biological process | Neutrophil chemotaxis | 9 | 0.00000031 |
| Biological process | Neutrophil migration | 9 | 0.00000098 |
| Biological process | Granulocyte chemotaxis | 9 | 0.0000014 |
| Biological process | Leukocyte migration | 13 | 0.0000033 |
| Biological process | Myeloid leukocyte migration | 10 | 0.0000042 |
| Biological process | Leukocyte chemotaxis | 10 | 0.000019 |
| Biological process | Cell chemotaxis | 11 | 0.000030 |
| Biological process | Regulation of granulocyte chemotaxis | 5 | 0.00040 |
| Biological process | Positive regulation of leukocyte migration | 7 | 0.00047 |
| Biological process | Leukocyte homeostasis | 6 | 0.0011 |
| Biological process | Positive regulation of neutrophil chemotaxis | 4 | 0.0012 |
| Biological process | Positive regulation of granulocyte chemotaxis | 4 | 0.0015 |
| Biological process | Cell migration | 20 | 0.0017 |
| Biological process | Regulation of neutrophil chemotaxis | 4 | 0.0020 |
| Biological process | Regulation of leukocyte migration | 7 | 0.0020 |
| Biological process | Positive regulation of neutrophil migration | 4 | 0.0021 |
| Biological process | Positive regulation of chemotaxis | 6 | 0.0033 |
| Biological process | Positive regulation of leukocyte chemotaxis | 5 | 0.0045 |
| Biological process | Positive regulation of defense response | 8 | 0.0054 |
| Biological process | Regulation of leukocyte chemotaxis | 5 | 0.0095 |
| Biological process | Positive regulation of cell migration | 9 | 0.020 |
| Biological process | Positive regulation of cell motility | 9 | 0.024 |
| Biological process | Positive regulation of cellular component movement | 9 | 0.027 |
| **Annotation cluster 4** | **Enrichment score: 3.24** | | |
| Biological process | Response to bacterium | 18 | 0.000013 |
| Biological process | Defense response to bacterium | 9 | 0.0021 |
| Biological process | Defense response to other organism | 12 | 0.0072 |
| **Annotation cluster 5** | **Enrichment score: 3.18** | |  |
| Biological process | Response to bacterium | 18 | 0.000013 |
| Biological process | Response to lipopolysaccharide | 11 | 0.00094 |
| Biological process | Cellular response to lipid | 13 | 0.0018 |
| Biological process | Cellular response to lipopolysaccharide | 8 | 0.0022 |
| Biological process | Cellular response to molecule of bacterial origin | 8 | 0.0027 |
| **Annotation cluster 6** | **Enrichment score: 3.16** | |  |
| Biological process | Leukocyte cell-cell adhesion | 18 | 0.00000030 |
| Biological process | Leukocyte aggregation | 16 | 0.0000029 |
| Biological process | Lymphocyte activation | 19 | 0.0000040 |
| Biological process | Hematopoietic or lymphoid organ development | 22 | 0.0000077 |
| Biological process | Regulation of leukocyte cell-cell adhesion | 12 | 0.000015 |
| Biological process | Immune system development | 22 | 0.000015 |
| Biological process | Lymphocyte proliferation | 12 | 0.000017 |
| Biological process | Hemopoiesis | 20 | 0.000042 |
| Biological process | T cell aggregation | 14 | 0.000052 |
| Biological process | Regulation of leukocyte activation | 14 | 0.000070 |
| Biological process | Regulation of lymphocyte activation | 13 | 0.000071 |
| Biological process | Positive regulation of leukocyte cell-cell adhesion | 9 | 0.000074 |
| Cellular component | MHC class II protein complex | 4 | 0.000077 |
| Biological process | Positive regulation of leukocyte activation | 11 | 0.000087 |
| Biological process | Positive regulation of cell activation | 11 | 0.000012 |
| Biological process | Antigen processing and presentation of exogenous peptide antigen via MHC class II | 4 | 0.00015 |
| Cellular component | External side of plasma membrane | 11 | 0.00017 |
| Biological process | External side of plasma membrane | 9 | 0.00022 |
| Biological process | Positive regulation of cell-cell adhesion | 10 | 0.00028 |
| Biological process | Regulation of T cell activation | 11 | 0.00059 |
| Biological process | Regulation of hemopoiesis | 8 | 0.00060 |
| Biological process | Positive regulation of hemopoiesis | 13 | 0.00089 |
| Biological process | Leukocyte differentiation | 9 | 0.00089 |
| Biological process | Positive regulation of lymphocyte activation | 8 | 0.0011 |
| Biological process | Regulation of lymphocyte proliferation | 4 | 0.0012 |
| Cellular component | MHC protein complex | 8 | 0.0012 |
| Biological process | Regulation of mononuclear cell proliferation | 7 | 0.0020 |
| Biological process | Positive regulation of T cell activation | 8 | 0.0025 |
| Biological process | T cell differentiation | 10 | 0.0027 |
| Biological process | Myeloid cell differentiation | 5 | 0.0041 |
| Biological process | Regulation of anion transport | 8 | 0.0048 |
| Biological process | Positive regulation of myeloid cell differentiation | 5 | 0.0053 |
| Biological process | Positive regulation of leukocyte differentiation | 6 | 0.0062 |
| Biological process | Response to interferon-gamma | 5 | 0.0086 |
| Biological process | Negative regulation of lymphocyte activation | 5 | 0.017 |
| Biological process | Regulation of myeloid cell differentiation | 6 | 0.021 |
| Cellular component | Plasma membrane protein complex | 9 | 0.023 |
| Biological process | Negative regulation of cell-cell adhesion | 5 | 0.027 |
| Biological process | Negative regulation of leukocyte activation | 5 | 0.028 |
| Biological process | Protein kinase B signaling | 5 | 0.036 |
| Biological process | Regulation of lymphocyte differentiation | 5 | 0.036 |
| Biological process | Negative regulation of T cell activation | 4 | 0.037 |
| Biological process | Positive regulation of protein kinase B signaling | 4 | 0.037 |
| Biological process | Negative regulation of cell activation | 5 | 0.040 |
| Biological process | Negative regulation of leukocyte cell-cell adhesion | 4 | 0.046 |
| **Annotation cluster 7** | **Enrichment score: 2.86** | |  |
| Biological process | Organ morphogenesis | 22 | 0.000034 |
| Biological process | Sensory organ morphogenesis | 9 | 0.0019 |
| Biological process | Sensory organ development | 10 | 0.042 |
| **Annotation cluster 8** | **Enrichment score: 2.51** | |  |
| Biological process | Acute inflammatory response | 8 | 0.000060 |
| Biological process | Positive regulation of inflammatory response | 7 | 0.00021 |
| Biological process | Positive regulation of humoral immune response | 3 | 0.0072 |
| Biological process | Regulation of acute inflammatory response | 4 | 0.011 |
| Biological process | Positive regulation of acute inflammatory response | 3 | 0.026 |
| Biological process | Activation of immune response | 7 | 0.033 |
| **Annotation cluster 9** | **Enrichment score: 2.43** | |  |
| Biological process | Leukocyte migration | 13 | 0.0000033 |
| Biological process | Regulation of secretion | 18 | 0.000053 |
| Biological process | Regulation of inflammatory response | 11 | 0.000065 |
| Biological process | Positive regulation of leukocyte cell-cell adhesion | 9 | 0.00007**4** |
| Biological process | Positive regulation of inflammatory response | 7 | 0.00021 |
| Biological process | Positive regulation of cell-cell adhesion | 9 | 0.00022 |
| Biological process | Cellular response to cytokine production | 14 | 0.00024 |
| Biological process | Positive regulation of secretion | 12 | 0.00041 |
| Biological process | Cytokine-mediated signaling pathway | 10 | 0.00085 |
| Biological process | Secretion | 19 | 0.0013 |
| Biological process | Positive regulation of secretion by cell | 10 | 0.0033 |
| Biological process | Positive regulation of intracellular signal transduction | 16 | 0.0036 |
| Biological process | Regulation of anion transport | 5 | 0.0041 |
| Biological process | Positive regulation of defense response | 8 | 0.0054 |
| Biological process | Regulation of secretion by cell | 13 | 0.0069 |
| Biological process | Positive regulation of homeostatic process | 7 | 0.0069 |
| Biological process | Positive regulation of transport | 16 | 0.0091 |
| Biological process | Regulation of peptide secretion | 7 | 0.0092 |
| Biological process | Regulation of peptide transport | 7 | 0.0096 |
| Molecular function | Monocarboxylic acid binding | 4 | 0.0099 |
| Biological process | Secretion by cell | 15 | 0.011 |
| Biological process | Positive regulation of ion transport | 7 | 0.012 |
| Biological process | ERK1 and ERK2 cascade | 7 | 0.015 |
| Biological process | Positive regulation of cell communication | 20 | 0.017 |
| Biological process | Peptide secretion | 7 | 0.022 |
| Biological process | Positive regulation of signal transduction | 18 | 0.023 |
| Biological process | Positive regulation of MAPK cascade | 9 | 0.026 |
| Biological process | Peptide transport | 7 | 0.028 |
| Biological process | Regulation of ion transport | 10 | 0.033 |
| Biological process | Amide transport | 7 | 0.035 |
| Biological process | Positive regulation of protein kinase B signaling | 4 | 0.037 |
| Biological process | Regulation of lipid transport | 4 | 0.038 |
| Biological process | Positive regulation of anion transport | 3 | 0.046 |
| Biological process | Icosanoid secretion | 3 | 0.049 |
| **Annotation cluster 10** | **Enrichment score: 2.3** | |  |
| Biological process | Positive regulation of leukocyte cell-cell adhesion | 9 | 0.000074 |
| Biological process | Positive regulation of cell-cell adhesion | 9 | 0.00022 |
| Biological process | Cellular extravasation | 4 | 0.0054 |
| Biological process | Positive regulation of cytokine production | 9 | 0.0073 |
| Biological process | Phagocytosis | 6 | 0.013 |
| Biological process | Leukocyte adhesion to vascular endothelial cell | 3 | 0.015 |
| Biological process | Endocytosis | 10 | 0.019 |
| Biological process | Regulation of phagocytosis | 4 | 0.026 |
| Biological process | Protein kinase B signaling | 5 | 0.036 |
| **Annotation cluster 11** | **Enrichment score: 2.14** | |  |
| Biological process | Animal organ development | 48 | 0.000026 |
| Biological process | Organ morphogenesis | 22 | 0.000034 |
| Molecular function | Sequence-specific DNA binding | 20 | 0.00019 |
| Biological process | Positive regulation of gene expression | 23 | 0.0092 |
| Biological process | Positive regulation of macromolecule metabolic process | 33 | 0.0093 |
| Biological process | Regulation of gene expression | 45 | 0.013 |
| Biological process | Positive regulation of macromolecule biosynthetic process | 21 | 0.015 |
| Biological process | Transcription, DNA-templated | 35 | 0.016 |
| Biological process | Positive regulation of cellular biosynthetic process | 21 | 0.033 |
| Biological process | Regulation of transcription, DNA-templated | 36 | 0.037 |
| Biological process | Regulation of RNA metabolic process | 37 | 0.037 |
| Biological process | Regulation of RNA biosynthetic process | 36 | 0.038 |
| Biological process | Regulation of macromolecule biosynthetic process | 40 | 0.042 |
| Biological process | Positive regulation of transcription, DNA-templated | 17 | 0.046 |
| Biological process | Positive regulation of RNA biosynthetic process | 17 | 0.047 |
| **Annotation cluster 12** | **Enrichment score: 2.13** | |  |
| Biological process | Regulation of inflammatory response | 11 | 0.000065 |
| Biological process | Positive regulation of apoptotic process | 10 | 0.032 |
| Biological process | Positive regulation of programmed cell death | 10 | 0.033 |
| Biological process | Positive regulation of cell death | 10 | 0.046 |
| **Annotation cluster 13** | **Enrichment score: 1.92** | |  |
| Biological process | Inflammatory response to antigenic stimulus | 5 | 0.0013 |
| Biological process | Negative regulation of growth of symbiont in host | 3 | 0.0099 |
| Biological process | Negative regulation of growth of symbiont involved in interaction with host | 3 | 0.0099 |
| Biological process | Regulation of growth of symbiont in host | 3 | 0.011 |
| Biological process | Modulation of growth of symbiont involved in interaction with host | 3 | 0.011 |
| Biological process | Growth of symbiont involved in interaction with host | 3 | 0.015 |
| Biological process | Regulation of cytokine biosynthetic process | 4 | 0.042 |
| Biological process | Cytokine biosynthetic process | 4 | 0.049 |
| **Annotation cluster 14** | **Enrichment score: 1.83** | |  |
| Biological process | Regulation of proteolysis | 12 | 0.0071 |
| Biological process | Proteolysis | 21 | 0.013 |
| Molecular function | Endopeptidase inhibitor activity | 6 | 0.017 |
| Biological process | Regulation of peptidase activity | 8 | 0.018 |
| Biological process | Negative regulation of proteolysis | 7 | 0.023 |
| **Annotation cluster 15** | **Enrichment score: 1.7** | |  |
| Biological process | Regulation of secretion by cell | 13 | 0.0069 |
| Biological process | Positive regulation of cytokine production | 9 | 0.0073 |
| Biological process | Negative regulation of secretion | 6 | 0.036 |
| Biological process | Regulation of endothelial cell proliferation | 4 | 0.038 |
| Biological process | Cytokine secretion | 5 | 0.047 |
| **Annotation cluster 16** | **Enrichment score: 1.5** | |  |
| Biological process | Response to estradiol | 5 | 0.018 |
| Biological process | Negative regulation of secretion | 6 | 0.036 |
| Biological process | Response to corticosteroid | 5 | 0.049 |
| **Annotation cluster 17** | **Enrichment score: 1.36** | |  |
| Biological process | Hair follicle development | 4 | 0.042 |
| Biological process | Hair cycle process | 4 | 0.044 |
| Biological process | Skin epidermis development | 4 | 0.045 |
|  |  |  |  |
| **DOWNREGULATED TRANSCRIPTS SINGLE MODERATE** | | | |
| **Functional classification** | **Gene Ontology Term** | **Number of genes** | **P-value** |
| **Annotation cluster 1** | **Enrichment score: 2.21** | | |
| Biological process | Atrial septum morphogenesis | 3 | 0.0013 |
| Biological process | Atrial septum development | 3 | 0.0020 |
| Biological process | Embryonic organ development | 7 | 0.0044 |
| Biological process | Cardiac atrium morphogenesis | 3 | 0.0051 |
| Biological process | Cardiac atrium development | 3 | 0.0064 |
| Biological process | Cardiac septum morphogenesis | 3 | 0.023 |
| Biological process | Organ morphogenesis | 8 | 0.043 |
| **Annotation cluster 2** | **Enrichment score: 1.83** | | |
| Biological process | Embryonic organ development | 7 | 0.0044 |
| Biological process | Labyrinthine layer development | 3 | 0.015 |
| Biological process | Embryonic placenta development | 3 | 0.048 |
