# Supplementary material for: Functional Analysis of the Cortical Transcriptome and Proteome Reveal Neurogenesis, Inflammation, and Cell Death after Repeated Traumatic Brain Injury In vivo
Source: Neurotrauma Rep. 2022 Jun 13;3(1):224–39. doi: 10.1089/neur.2021.0059 (PMC9279125; doi:10.1089/neur.2021.0059)
Supplement: Supplemental data [file Suppl_TableS9.docx]

**Supplemental table 9:** Functional annotation clustering results for transcripts which had their expression levels significantly changed after double mild traumatic brain injuries. Gene Ontology terms based on biological processes, cellular components, and molecular functions sharing gene members and functions were clustered through DAVID. Data shows the number of encoding genes associated with each term, while p-values derived from EASE-scores demonstrate the gene enrichment in the annotated terms.

| UPREGULATED TRANSCRIPTS DOUBLE MILD | | | |
| --- | --- | --- | --- |
| Functional classification | Gene Ontology Term | Number of genes | **P-value** |
| Annotation cluster 1 | Enrichment score: 2.41 | | |
| Molecular function | Serine-type endopeptidase activity | 7 | 0.00092 |
| Molecular function | Serine-type peptidase activity | 7 | 0.0016 |
| Molecular function | Endopeptidase | 7 | 0.042 |
| **Annotation cluster 2** | **Enrichment score: 1.91** | | |
| Biological process | Positive regulation of cytokine production | 8 | 0.0057 |
| Biological process | Positive regulation of phosphorus metabolic process | 13 | 0.018 |
| Biological process | Positive regulation of phosphate metabolic process | 13 | 0.018 |
| **Annotation cluster 3** | **Enrichment score: 1.82** | | |
| Biological process | Regulation of amine transport | 4 | 0.0090 |
| Biological process | Amine transport | 4 | 0.010 |
| Biological process | Positive regulation of secretion | 7 | 0.037 |
| **Annotation cluster 4** | **Enrichment score: 1.6** | | |
| Biological process | Regulation of blood vessel size | 5 | 0.016 |
| Biological process | Regulation of vasculature development | 6 | 0.017 |
| Biological process | Regulation of blood pressure | 5 | 0.023 |
| Biological process | Regulation of vasodilation | 3 | 0.035 |
| Biological process | Regulation of angiogenesis | 5 | 0.047 |
| **Annotation cluster 5** | **Enrichment score: 1.5** | |  |
| Biological process | Regulation of phagocytosis | 4 | 0.013 |
| Biological process | Regulation of endocytosis | 5 | 0.039 |
| Biological process | Positive regulation of phagocytosis | 3 | 0.044 |
| Biological process | Regulation of vesicle-mediated transport | 7 | 0.047 |
|  |  |  |  |
| **DOWNREGULATED TRANSCRIPTS DOUBLE MILD** | | | |
| **Functional classification** | **Gene Ontology Term** | **Number of genes** | **P-value** |
| **Annotation cluster 1** | **Enrichment score: 2.06** | | |
| Biological process | Neutrophil chemotaxis | 5 | 0.00066 |
| Biological process | Neutrophil migration | 5 | 0.0012 |
| Biological process | Granulocyte chemotaxis | 5 | 0.0014 |
| Biological process | Leukocyte migration | 7 | 0.0026 |
| Biological process | Myeloid leukocyte migration | 5 | 0.0065 |
| Biological process | Positive regulation of neutrophil chemotaxis | 3 | 0.0071 |
| Biological process | Positive regulation of leukocyte chemotaxis | 4 | 0.0075 |
| Biological process | Regulation of leukocyte migration | 5 | 0.0078 |
| Biological process | Positive regulation of granulocyte chemotaxis | 3 | 0.0082 |
| Biological process | Regulation of neutrophil chemotaxis | 3 | 0.0098 |
| Biological process | Positive regulation of neutrophil migration | 3 | 0.010 |
| Biological process | Leukocyte chemotaxis | 5 | 0.012 |
| Biological process | Regulation of leukocyte chemotaxis | 4 | 0.013 |
| Biological process | Regulation of granulocyte chemotaxis | 3 | 0.019 |
| Biological process | Regulation of cell migration | 9 | 0.019 |
| Biological process | Positive regulation of leukocyte migration | 4 | 0.021 |
| Biological process | Positive regulation of chemotaxis | 4 | 0.022 |
| Biological process | Regulation fo cell motility | 9 | 0.025 |
| Biological process | Cell chemotaxis | 5 | 0.031 |
| Biological process | Cell migration | 11 | 0.045 |
| **Annotation cluster 2** | **Enrichment score: 1.76** | | |
| Biological process | Protein secretion | 8 | 0.0087 |
| Biological process | Cytokin secretion | 5 | 0.0088 |
| Biological process | Positive regulation of secretion by cell | 7 | 0.0095 |
| Biological process | Positive regulation of secretion | 7 | 0.013 |
| Biological process | Regulation of secretion by cell | 9 | 0.014 |
| Biological process | Positive regulation of cytokine secretion | 4 | 0.014 |
| Biological process | Regulation fo secretion | 9 | 0.021 |
| Biological process | Positive regulation of protein secretion | 5 | 0.025 |
| Biological process | Secretion by cell | 10 | 0.026 |
| Biological process | Regulation of cytokine secretion | 4 | 0.038 |
| Biological process | Regulation of protein secretion | 6 | 0.047 |
