# Supplementary material for: Functional Analysis of the Cortical Transcriptome and Proteome Reveal Neurogenesis, Inflammation, and Cell Death after Repeated Traumatic Brain Injury In vivo
Source: Neurotrauma Rep. 2022 Jun 13;3(1):224–39. doi: 10.1089/neur.2021.0059 (PMC9279125; doi:10.1089/neur.2021.0059)
Supplement: Supplemental data [file Suppl_TableS11.docx]

**Supplemental table 11:** Functional annotation clustering results for transcripts which had their expression levels significantly changed after double moderate traumatic brain injuries. Gene Ontology terms based on biological processes, cellular components, and molecular functions sharing gene members and functions were clustered through DAVID. Data shows the number of encoding genes associated with each term, while p-values derived from EASE-scores demonstrate the gene enrichment in the annotated terms.

| UPREGULATED TRANSCRIPTS DOUBLE MODERATE | | | |
| --- | --- | --- | --- |
| Functional classification | Gene Ontology Term | Number of genes | **P-value** |
| Annotation cluster 1 | Enrichment score: 3.03 | | |
| Cell component | Plasma membrane part | 60 | 0.00015 |
| Cell component | Intrinsic component of plasma membrane | 36 | 0.0014 |
| Cell component | Integral component of plasma membrane | 33 | 0.0040 |
| **Annotation cluster 2** | **Enrichment score: 2.97** | | |
| Biological process | Cell migration | 33 | 0.000063 |
| Biological process | Regulation of cell motility | 25 | 0.000070 |
| Biological process | Regulation of cell migration | 24 | 0.000086 |
| Biological process | Positive regulation of cell migration | 13 | 0.013 |
| Biological process | Positive regulation of cell motility | 13 | 0.016 |
| Biological process | Positive regulation of cellular component movement | 13 | 0.019 |
| **Annotation cluster 3** | **Enrichment score: 2.56** | | |
| Biological process | Anion transport | 16 | 0.00038 |
| Biological process | Organic anion transport | 11 | 0.0050 |
| Biological process | Amino acid transport | 6 | 0.011 |
| **Annotation cluster 4** | **Enrichment score: 2.46** | | |
| Biological process | Blood vessel development | 21 | 0.00023 |
| Biological process | Vasculature development | 21 | 0.00047 |
| Biological process | Regulation of vasculature development | 11 | 0.0019 |
| Biological process | Positive regulation of vasculutre development | 8 | 0.0035 |
| Biological process | Cardiovascular system development | 25 | 0.0050 |
| Biological process | Circulatory system development | 25 | 0.0050 |
| Biological process | Regulation of angiogenesis | 9 | 0.011 |
| Biological process | Angiogenesis | 13 | 0.012 |
| Biological process | Positive regulation of angiogenesis | 6 | 0.030 |
| **Annotation cluster 5** | **Enrichment score: 2.38** | |  |
| Biological process | Positive regulation of inflammatory response | 8 | 0.00060 |
| Biological process | Positive regulation of defense response | 10 | 0.010 |
| Biological process | Regulation of inflammatory response | 10 | 0.012 |
| **Annotation cluster 6** | **Enrichment score: 2.37** | |  |
| Biological process | Eye development | 14 | 0.0022 |
| Biological process | Sensory organ development | 17 | 0.0054 |
| Biological process | Camera-type eye development | 12 | 0.0064 |
| **Annotation cluster 7** | **Enrichment score: 1.88** | |  |
| Biological process | Carbohydrate homeostasis | 11 | 0.0016 |
| Biological process | Cellular glucose homeostasis | 7 | 0.014 |
| Biological process | Cellular chemical homeostasis | 18 | 0.019 |
| Biological process | Response to monosaccharide | 8 | 0.022 |
| Biological process | Cellular response to carbohydrate stimulus | 6 | 0.041 |
| **Annotation cluster 8** | **Enrichment score: 1.79** | |  |
| Biological process | Cell development | 45 | 0.0033 |
| Biological process | Regulation of nervous system development | 22 | 0.0078 |
| Biological process | Neurogensis | 33 | 0.011 |
| Biological process | Central nervous system development | 22 | 0.013 |
| Biological process | Positive regulation of neurogenesis | 13 | 0.025 |
| Biological process | Nervous system development | 40 | 0.032 |
| Biological process | Regulation of neurogenesis | 18 | 0.037 |
| Biological process | Positive regulation of cell development | 14 | 0.044 |
| **Annotation cluster 9** | **Enrichment score: 1.74** | |  |
| Biological process | Negative regulation of viral genome replication | 5 | 0.0020 |
| Biological process | Regulation of viral genome replication | 5 | 0.016 |
| Biological process | Negative regulation of viral life cycle | 5 | 0.019 |
| Biological process | Viral genome replication | 5 | 0.025 |
| Biological process | Viral life cycle | 8 | 0.027 |
| Biological process | Negative regulation of viral process | 5 | 0.039 |
| Biological process | Regulation of viral life cycle | 6 | 0.040 |
| **Annotation cluster 10** | **Enrichment score: 1.61** | |  |
| Biological process | Tube morphogenesis | 13 | 0.0072 |
| Biological process | Epithelial tube morphogenesis | 12 | 0.0084 |
| Biological process | Morphogenesis of a branching epithelium | 8 | 0.022 |
| Biological process | Neural tube development | 7 | 0.035 |
| Biological process | Morphogenesis of an epithelium | 13 | 0.045 |
| Biological process | Neural tube closure | 5 | 0.048 |
| Biological process | Tube closure | 5 | 0.049 |
| **Annotation cluster 11** | **Enrichment score: 1.61** | |  |
| Biological process | Regulation fo cellular carbohydrate metabolic process | 7 | 0.014 |
| Biological process | Negative regulation of cellular carbohydrate metabolic process | 4 | 0.028 |
| Biological process | Negative regulation of carbohydrate metabolic process | 4 | 0.040 |
| **Annotation cluster 12** | **Enrichment score: 1.61** | |  |
| Biological process | ERK1 and ERK2 cascade | 10 | 0.0072 |
| Biological process | Regulation of phosphate metabolic process | 32 | 0.017 |
| Biological process | Positive regulation of intracellular signal transduction | 20 | 0.024 |
| Biological process | Regulation of intracellular signal transduction | 30 | 0.026 |
| Biological process | Positive regulation of phosphate metabolic process | 22 | 0.030 |
| Biological process | Positive regulation of phosphorus metabolic process | 22 | 0.030 |
| Biological process | Regulation of protein metabolic process | 43 | 0.035 |
| Biological process | Negative regulation of protein metabolic process | 21 | 0.037 |
| Biological process | Regulation of protein modification process | 30 | 0.041 |
| Biological process | Regulation of signal transduction | 42 | 0.047 |
|  |  |  |  |
| **DOWNREGULATED TRANSCRIPTS DOUBLE MODERATE** | | | |
| **Functional classification** | **Gene Ontology Term** | **Number of genes** | **P-value** |
| **Annotation cluster 1** | **Enrichment score: 2.07** | | |
| Cellular component | Plasma membrane region | 16 | 0.0012 |
| Cellular component | Postsynaptic membrane | 6 | 0.013 |
| Cellular component | Synaptic membrane | 6 | 0.039 |
| **Annotation cluster 2** | **Enrichment score: 2.04** | | |
| Biological process | Regulation of meiotic cell cylce | 4 | 0.0037 |
| Biological process | Negative regulation of meiotic cell cycle | 3 | 0.0083 |
| Biological process | Regulation of nuclear division | 5 | 0.026 |
| **Annotation cluster 3** | **Enrichment score: 1.96** | | |
| Biological process | Regulation of meiotic cell cycle | 4 | 0.0037 |
| Biological process | Nuclear division | 11 | 0.0047 |
| Biological process | Meiotic nuclear division | 6 | 0.0073 |
| Biological process | Meiotic cell cycle process | 6 | 0.0094 |
| Biological process | Nuclear chromosome segregation | 6 | 0.020 |
| Biological process | Meiotic chromosome segregation | 4 | 0.021 |
| Biological process | Meiosis I | 4 | 0.035 |
| **Annotation cluster 4** | **Enrichment score: 1.96** | | |
| Cellular component | Plasma membrane region | 16 | 0.0012 |
| Cellular component | Integral component of plasma membrane | 18 | 0.0032 |
| Cellular component | Plasma membrane part | 27 | 0.0052 |
| Cellular component | Intrinsic component of plasma membrane | 18 | 0.0054 |
| Cellular component | Transmembrane transport | 15 | 0.0061 |
| Cellular component | Ion channel activity | 7 | 0.025 |
| Cellular component | Substrate-specific channel activity | 7 | 0.028 |
| Cellular component | Ion transport | 16 | 0.032 |
| Cellular component | Ion transmembrane transport | 10 | 0.038 |
| Cellular component | Cation transmembrane transporter | 8 | 0.040 |
| **Annotation cluster 5** | **Enrichment score: 1.37** | |  |
| Biological process | Establishment of synaptic vesicle | 4 | 0.041 |
| Biological process | Synaptic vesicle transport | 4 | 0.041 |
| Biological process | Synaptic vesicle localization | 4 | 0.048 |
