# Supplementary material for: Functional Analysis of the Cortical Transcriptome and Proteome Reveal Neurogenesis, Inflammation, and Cell Death after Repeated Traumatic Brain Injury In vivo
Source: Neurotrauma Rep. 2022 Jun 13;3(1):224–39. doi: 10.1089/neur.2021.0059 (PMC9279125; doi:10.1089/neur.2021.0059)
Supplement: Supplemental data [file Suppl_Text.docx]

**Supplemental Results**

*Angiogenesis*

Biological processes-related GO terms associated with the regulation of angiogenesis and blood vessel and vasculature development were only observed among upregulated transcripts in the MiD, MoS, and MoD conditions. For MiD, 4 terms associated with these processes were identified, including regulation of blood vessel size and blood pressure (Supplemental T3), and comprising 4.8% (9 transcripts) of all upregulated transcripts (Fig. 4c). After a MoS TBI, 7% (16 transcripts) of upregulated transcripts were categorized in 7 angiogenesis-related terms (Fig. 4b, Supplemental T2). In the MoD condition, nine angiogenesis-related GO terms were observed, including circulatory and cardiovascular system development in addition to the previously mentioned (Supplementary T4), which comprised 7.4% (25 transcripts) of upregulated transcripts in this group (Fig. 4d). Regarding functional clustering, clusters including the above-mentioned biological processes were also identified for upregulated transcripts in the three groups, with an enrichment score of 3.66 for MoS, 2.46 for MoD, and 1.6 for MiD (Tables 2-4).

*Cell communication*

Biological processes involved in signal transduction, protein secretion and synaptic vesicle transport were also observed at the protein and transcript level. Every condition had upregulated transcripts associated with these terms (Fig. 4a-d), and MiD, MoS and MoD conditions also showed downregulated transcripts involving cell communication (Fig. 4E-G). Regarding upregulated cell communication processes from the transcriptome, MoS had the most with 20 different biological processes and MiD had the most downregulated categories with 8 (Supplementary T2 and T3). Looking at the proteome data, all conditions except MiS had at least one process upregulated and the double conditions showed downregulated processes. MoD had the most processes (10) from the proteome data involved in cell communication (Supplementary T4). Interestingly, the biological process “positive regulation of transport” was upregulated in every condition in either the proteome or transcriptome data. Some of the most notable genes found across these groups in “positive regulation of transport” include arginine vasopressin, oxytocin, interleukin 1 alpha, leptin and complement component 3 (Supplemental F1).

*Protein metabolism and modifications*

When observing the proteome data, every group had at least one significant biological process upregulated dealing with protein modifications (Fig. 4a-d). The MiD condition had four significant processes specific to proteolysis and catabolism upregulated while the MiS and MoS conditions had none (Supplementary T1-3). Interestingly, in the MoS condition four processes were involved in proteolysis and catabolism and significantly downregulated. When analyzing the transcriptome, the MoS condition had two processes involving proteolysis downregulated, similar to the proteome data, and three processes upregulated involving proteolysis, contrary to the proteome data.
