# Supplementary material for: Functional Analysis of the Cortical Transcriptome and Proteome Reveal Neurogenesis, Inflammation, and Cell Death after Repeated Traumatic Brain Injury In vivo
Source: Neurotrauma Rep. 2022 Jun 13;3(1):224–39. doi: 10.1089/neur.2021.0059 (PMC9279125; doi:10.1089/neur.2021.0059)
Supplement: Supplemental data [file Suppl_TableS5.docx]

**Supplemental table 5:** Functional annotation clustering results for transcripts which had their expression levels significantly changed after a single mild traumatic brain injury. Gene Ontology terms based on biological processes, cellular components, and molecular functions sharing gene members and functions were clustered through DAVID. Data shows the number of encoding genes associated with each term, while p-values derived from EASE-scores demonstrate the gene enrichment in the annotated terms.

| UPREGULATED TRANSCRIPTS SINGLE MILD | | | |
| --- | --- | --- | --- |
| Functional classification | Gene Ontology Term | Number of genes | **P-value** |
| Annotation cluster 1 | Enrichment score: 2.79 | | |
| Biological process | Ion homeostasis | 13 | 0.00035 |
| Biological process | Cellular ion homeostasis | 11 | 0.0011 |
| Biological process | Cellular chemical homeostasis | 12 | 0.0019 |
| Biological process | Positive regulation of ion transport | 6 | 0.0097 |
| **Annotation cluster 2** | **Enrichment score: 2.01** | | |
| Biological process | Positive regulation of anion transport | 4 | 0.0016 |
| Biological process | Positive regulation of ion transport | 6 | 0.0097 |
| Biological process | Regulation of anion transport | 4 | 0.010 |
| Biological process | Anion transport | 7 | 0.023 |
| Biological process | Regulation of sensory perception | 3 | 0.025 |
