# Supplementary material for: Functional Analysis of the Cortical Transcriptome and Proteome Reveal Neurogenesis, Inflammation, and Cell Death after Repeated Traumatic Brain Injury In vivo
Source: Neurotrauma Rep. 2022 Jun 13;3(1):224–39. doi: 10.1089/neur.2021.0059 (PMC9279125; doi:10.1089/neur.2021.0059)
Supplement: Supplemental data [file Suppl_TableS8.docx]

**Supplemental table 8:** Functional annotation clustering results for proteins which had their expression levels significantly changed after a single moderate traumatic brain injury. Gene Ontology terms based on biological processes, cellular components, and molecular functions sharing gene members and functions were clustered through DAVID. Data shows the number of encoding genes associated with each term, while p-values derived from EASE-scores demonstrate the gene enrichment in the annotated terms.

| UPREGULATED PROTEINS SINGLE MODERATE | | | |
| --- | --- | --- | --- |
| Functional classification | Gene Ontology Term | Number of genes | **P-value** |
| Annotation cluster 1 | Enrichment score: 2.71 | | |
| Biological process | Establishment of protein localization to plasma membrane | 7 | 0.00043 |
| Biological process | Protein localization to plasma membrane | 7 | 0.0033 |
| Biological process | Protein localization to cell periphery | 7 | 0.0052 |
| **Annotation cluster 2** | **Enrichment score: 2.67** | | |
| Biological process | Actin filament depolymerization | 5 | 0.00057 |
| Biological process | Negative regulation of protein depolymerization | 5 | 0.00096 |
| Biological process | Negative regulation of protein polymerization | 5 | 0.0011 |
| Biological process | Negative regulation of protein complex disassembly | 5 | 0.0011 |
| Biological process | Regulation of protein depolymerization | 5 | 0.0022 |
| Biological process | Negative regulation of protein complex assembly | 5 | 0.010 |
| Biological process | Negative regulation of cytoskeleton organization | 5 | 0.013 |
| **Annotation cluster 3** | **Enrichment score: 2.5** | | |
| Biological process | Regulation of actin polymerization or depolymerization | 7 | 0.0012 |
| Biological process | Regulation of actin filament length | 7 | 0.0013 |
| Biological process | Regulation of actin cytoskeleton organization | 7 | 0.020 |
| **Annotation cluster 4** | **Enrichment score: 2.27** | | |
| Biological process | Regulation of actin filament polymerization | 6 | 0.0043 |
| Biological process | Actin filament polymerization | 6 | 0.0042 |
| Biological process | Regulation of protein polymerization | 6 | 0.0092 |
| **Annotation cluster 5** | **Enrichment score: 1.56** | |  |
| Cellular component | Sarcomere | 6 | 0.022 |
| Cellular component | Contractile fiber part | 6 | 0.028 |
| Cellular component | Myofibril | 6 | 0.033 |
|  |  |  |  |
| **DOWNREGULATED PROTEINS SINGLE MODERATE** | | | |
| **Functional classification** | **Gene Ontology Term** | **Number of genes** | **P-value** |
| **Annotation cluster 1** | **Enrichment score: 5.17** | | |
| Biological process | Methylglyoxal catabolic process | 4 | 0.0000023 |
| Biological process | Meythylglyoxal metabolic process | 4 | 0.0000041 |
| Biological process | Ketone catabolic process | 4 | 0.000032 |
| **Annotation cluster 2** | **Enrichment score: 3.49** | | |
| Cellular component | Proteasome core complex | 4 | 0.00021 |
| Molecular function | Threonine-type peptidase activity | 4 | 0.00023 |
| Biological process | Antigen processing and presentation of exogenous peptide antigen via MHC class 1 | 4 | 0.00069 |
| **Annotation cluster 3** | **Enrichment score: 1.71** | | |
| Biological process | Regulation of ion transmembrane transporter activity | 5 | 0.015 |
| Biological process | Regulation of transmembrane transporter activity | 5 | 0.017 |
| Biological process | Regulation of transporter activity | 5 | 0.0 |
| Biological process | Regulation of cation transmembrane transport | 5 | 0.028 |
| **Annotation cluster 4** | **Enrichment score: 1.56** | | |
| Cellular component | Sarcomere | 5 | 0.022 |
| Cellular component | Contractile fiber part | 5 | 0.021 |
| Cellular component | Myofibril | 5 | 0.050 |
| Cellular component | Contractile fiber | 5 | 0.032 |
