# Supplementary material for: Functional Analysis of the Cortical Transcriptome and Proteome Reveal Neurogenesis, Inflammation, and Cell Death after Repeated Traumatic Brain Injury In vivo
Source: Neurotrauma Rep. 2022 Jun 13;3(1):224–39. doi: 10.1089/neur.2021.0059 (PMC9279125; doi:10.1089/neur.2021.0059)
Supplement: Supplemental data [file Suppl_TableS10.docx]

**Supplemental table 10:** Functional annotation clustering results for proteins which had their expression levels significantly changed after double mild traumatic brain injuries. Gene Ontology terms based on biological processes, cellular components, and molecular functions sharing gene members and functions were clustered through DAVID. Data shows the number of encoding genes associated with each term, while p-values derived from EASE-scores demonstrate the gene enrichment in the annotated terms.

| UPREGULATED PROTEINS DOUBLE MILD | | | |
| --- | --- | --- | --- |
| Functional classification | Gene Ontology Term | Number of genes | **P-value** |
| Annotation cluster 1 | Enrichment score: 2.7 | | |
| Cellular component | Respiratory chain complex 1 | 4 | 0.0014 |
| Cellular component | Mitochondrial respiratory chain complex 1 | 4 | 0.0014 |
| Cellular component | NADH dehydrogenase complex | 4 | 0.0014 |
| Cellular component | Mitochondrial respiratory chain | 4 | 0.0062 |
| **Annotation cluster 2** | **Enrichment score: 2.63** | | |
| Biological process | Purine nucleotide metabolic process | 9 | 0.00067 |
| Biological process | Nucleotide metabolic process | 9 | 0.0029 |
| Biological process | Nucleoside phosphate metabolic process | 9 | 0.0032 |
| Biological process | Nucleobase-containing small molecule metabolic process | 9 | 0.0052 |
| **Annotation cluster 3** | **Enrichment score: 2.2** | | |
| Cellular component | Inner mitochondrial membrane protein complex | 5 | 0.0027 |
| Cellular component | Mitochondrial protein complex | 5 | 0.0067 |
| Cellular component | Mitochondrial membrane part | 5 | 0.0014 |
| **Annotation cluster 4** | **Enrichment score: 2.09** | | |
| Biological process | Proteolysis involved in cellular protein catabolic process | 9 | 0.0037 |
| Biological process | Cellular protein catabolic process | 9 | 0.0049 |
| Biological process | Protein catabolic process | 9 | 0.0013 |
| Biological process | Cellular macromolecule catabolic process | 9 | 0.0019 |
| **Annotation cluster 5** | **Enrichment score: 1.72** | | |
| Molecular function | Purine ribonucleoside binding | 16 | 0.016 |
| Molecular function | Purine ribonucleotide binding | 16 | 0.019 |
| Molecular function | Purine nucleotide binding | 16 | 0.020 |
| Molecular function | Ribonucleotide binding | 16 | 0.021 |
| **Annotation cluster 6** | **Enrichment score: 1.6** | | |
| Biological process | Purine-containing compound biosynthetic process | 5 | 0.019 |
| Biological process | Ribose phosphate biosynthetic process | 5 | 0.019 |
| Biological process | Nucleotide biosynthetic process | 5 | 0.032 |
| Biological process | Nucleoside phosphate biosynthetic process | 5 | 0.034 |
| **Annotation cluster 7** | **Enrichment score: 1.58** | | |
| Biological process | Cerebral cortex cell migration | 3 | 0.018 |
| Biological process | Telencephalon cell migration | 3 | 0.030 |
| Biological process | Forebrain cell migration | 3 | 0.032 |
| **Annotation cluster 8** | **Enrichment score: 1.41** | | |
| Molecular function | ATP binding | 13 | 0.034 |
| Molecular function | Adenyl ribonucleiotide binding | 13 | 0.041 |
| Molecular function | Adenyl nucleotide binding | 13 | 0.042 |
|  |  |  |  |
| **DOWNREGULATED PROTEINS DOUBLE MILD** | | | |
| **Functional classification** | **Gene Ontology Term** | **Number of genes** | **P-value** |
| **Annotation cluster 1** | **Enrichment score: 2.22** | | |
| Biological process | Nucleoside triphosphate biosynthetic process | 4 | 0.0033 |
| Biological process | Nucleoside monophosphate biosynthetic process | 4 | 0.0048 |
| Biological process | Nucleoside biosynthetic process | 4 | 0.0088 |
| Biological process | Glycosyl compound biosynthetic process | 4 | 0.0093 |
| **Annotation cluster 2** | **Enrichment score: 2.06** | | |
| Biological process | Nucleotide metabolic process | 8 | 0.0072 |
| Biological process | Nucleoside phosphate metabolic process | 8 | 0.0078 |
| Biological process | Necluobase-contaning small molecule metabolic process | 8 | 0.012 |
| **Annotation cluster 3** | **Enrichment score: 1.96** | | |
| Biological process | Nucleoside monophosphate metabolic process | 6 | 0.0028 |
| Biological process | Ribonucleoside metabolic process | 6 | 0.0066 |
| Biological process | Nucleoside metabolic process | 6 | 0.0085 |
| Biological process | Ribonucleotide metabolic process | 6 | 0.031 |
| Biological process | Ribose phosphate metabolic process | 6 | 0.034 |
| **Annotation cluster 4** | **Enrichment score: 1.93** | | |
| Biological process | Establishment of protein localization to mitochondrial membrane | 3 | 0.0011 |
| Biological process | Intracellular protein transmembrane import | 3 | 0.013 |
| Biological process | Mitochondrial transmembrane transport | 3 | 0.013 |
| Biological process | Intracellular protein transmembrane transport | 3 | 0.018 |
| Biological process | Protein transmembrane transport | 3 | 0.019 |
| Biological process | Mitochondrial membrane organization | 3 | 0.041 |
| **Annotation cluster 5** | **Enrichment score: 1.6** | |  |
| Biological process | Regulation of actin filament polymerization | 4 | 0.020 |
| Biological process | Actin filament polymerization | 4 | 0.020 |
| Biological process | Regulation of actin polymerization or depolymerization | 4 | 0.027 |
| Biological process | Regulation fo actin filament length | 4 | 0.028 |
| Biological process | Regulation fo protein polymerization | 4 | 0.033 |
