# Supplementary material for: Functional Analysis of the Cortical Transcriptome and Proteome Reveal Neurogenesis, Inflammation, and Cell Death after Repeated Traumatic Brain Injury In vivo
Source: Neurotrauma Rep. 2022 Jun 13;3(1):224–39. doi: 10.1089/neur.2021.0059 (PMC9279125; doi:10.1089/neur.2021.0059)
Supplement: Supplemental data [file Suppl_TableS12.docx]

**Supplemental table 12:** Functional annotation clustering results for proteins which had their expression levels significantly changed after double moderate traumatic brain injuries. Gene Ontology terms based on biological processes, cellular components, and molecular functions sharing gene members and functions were clustered through DAVID. Data shows the number of encoding genes associated with each term, while p-values derived from EASE-scores demonstrate the gene enrichment in the annotated terms.

| UPREGULATED PROTEINS DOUBLE MODERATE | | | |
| --- | --- | --- | --- |
| Functional classification | Gene Ontology Term | Number of genes | **P-value** |
| Annotation cluster 1 | Enrichment score: 3.16 | | |
| Cellular component | Integral component of mitochondrial membrane | 5 | 0.00019 |
| Cellular component | Intrinsic component of mitochondrial membrane | 5 | 0.00022 |
| Cellular component | Integral component of organelle membrane | 5 | 0.0082 |
| **Annotation cluster 2** | **Enrichment score: 2.41** | | |
| Cellular component | MICOS complex | 3 | 0.00038 |
| Biological process | Inner mitochondrial membrane organization | 3 | 0.0036 |
| Biological process | Mitochondrial membrane organization | 3 | 0.044 |
| **Annotation cluster 3** | **Enrichment score: 1.75** | | |
| Biological process | Positive regulation of receptor internalization | 3 | 0.0050 |
| Biological process | Regulation of receptor internalization | 3 | 0.011 |
| Biological process | Positive regulation of receptor-mediated endocystosis | 3 | 0.020 |
| Biological process | Receptor internalization | 3 | 0.041 |
| Biological process | Regulation of receptor-mediated endocytosis | 3 | 0.045 |
| **Annotation cluster 4** | **Enrichment score: 1.62** | | |
| Cellular component | NADH dehydrogenase complex | 3 | 0.024 |
| Cellular component | Mitochondrial respiratory chain complex 1 | 3 | 0.024 |
| Cellular component | Respiratory chain complex 1 | 3 | 0.024 |
